# Phytotherapeutic Analysis of Chloroform-Based Fractions of *Alstonia scholaris* and *Wrightia tinctoria* Extracts Reveals Potent Anti-Psoriatic Activity: An In Vitro and In Vivo Study

**DOI:** 10.3390/ph18030304

**Published:** 2025-02-22

**Authors:** Madhavi Ojha, Nilanshu Manocha, Vinod Kumar, Ganeshan Karthikeyan, Devinder Toor

**Affiliations:** 1 Amity Institute of Virology and Immunology, Amity University Uttar Pradesh, Sector-125, Noida 201313, Uttar Pradesh, India; km.ojha@s.amity.edu (M.O.); karanarohan@gmail.com (G.K.); 2 Magan Centre for Applied Mycology, Faculty of Engineering and Applied Sciences, Cranfield University, Cranfield MK43 0AL, UK; vinod.kumar@cranfield.ac.uk

**Keywords:** psoriasis, medicinal plants, *Alstonia scholaris*, *Wrightia tinctoria*, HaCaT cells, keratinocytes

## Abstract

**Background/Objectives:** Psoriasis, a prevalent dermatological disorder, poses therapeutic challenges due to limited effective treatments or adverse side-effects. Traditional medicinal plants like *Alstonia scholaris* and *Wrightia tinctoria*, historically used in Ayurvedic and Siddha practices, show potential in treating inflammatory skin diseases. This study aims to explore their in vitro and in vivo anti-psoriatic properties to develop safer and more effective therapies. **Methods**: Chloroform:methanol fractions from ethanol extracts of *Alstonia scholaris* and *Wrightia tinctoria* were evaluated for anti-psoriatic activity. In vitro assays using HaCaT cells assessed cell viability, apoptosis, and inflammatory markers. In vivo studies utilized an IMQ-induced psoriasis mouse model, evaluating skin lesions, histopathology, and cytokine profiles. **Results**: Chloroform fractions significantly reduced HaCaT cell viability and induced apoptosis. They also dose-dependently downregulated IL-8 and RANTES levels. In vivo, these fractions reduced skin inflammation, edema, and psoriasis scores. Histopathological analysis showed decreased epidermal thickness and dermal inflammation. Key psoriasis biomarkers IL-17 and IL-23 were significantly reduced. **Conclusions**: Chloroform:methanol fractions from *Alstonia scholaris* and *Wrightia tinctoria* demonstrated potent anti-psoriatic effects in vitro and in vivo. These findings support their potential as novel phytotherapeutic agents for managing psoriasis, offering promise for further development and clinical application.

## 1. Introduction

Psoriasis is a chronic, immune-mediated skin disorder that affects over 2% of the global population [1]. It is primarily characterized by the presence of erythematous plaques covered with silvery scales, which can lead to symptoms like itching, pain, and discomfort. In addition to its cutaneous manifestations, psoriasis is often associated with various comorbidities, including metabolic syndrome, non-alcoholic fatty liver disease, psoriatic arthritis, and psychological disorders such as anxiety and depression [2]. The disease’s pathogenesis involves a complex interplay of genetic and environmental factors, leading to the hyper-proliferation of keratinocytes, immune dysregulation, and the infiltration of pro-inflammatory cells in the skin [3]. The immune system plays a pivotal role in psoriasis, with cytokines such as tumour necrosis factor alpha (TNF-α), interleukin (IL) -6, IL-8, IL-17, IL-22, IL-23, and RANTES (Regulated upon activation, normal T Cell expressed and presumably secreted) being central to the inflammatory processes observed in the disease [4,5].

The therapeutic strategies for psoriasis mainly include topical corticosteroids, phototherapy, systemic medications, and biologics. However, these treatments are often associated with significant side effects, such as cutaneous atrophy, organ toxicity, broad-spectrum immunosuppression, and challenges related to patient compliance and long-term safety [6,7,8]. For instance, roflumilast, a repurposed drug for the topical treatment of plaque psoriasis, inhibits phosphodiesterase-4, an enzyme that degrades cAMP, thereby enhancing anti-inflammatory responses. Recent phase II trials have shown its high efficacy; however, transient gastrointestinal symptoms, weight loss, headaches, and insomnia have been reported as adverse effects [9]. Biologic therapies have improved disease management, though they come with risks of immunogenicity, infections, and organ toxicity, with patients often developing resistance over time and long-term safety data still being insufficient [10,11,12]. Additionally, the high cost of biologics and their limited accessibility in certain regions further hinder effective treatment [13]. Varying treatment responses among patients, particularly in intertriginous or difficult-to-treat areas like the scalp and nails, complicate disease management [12]. These challenges highlight the urgent need for safer, more effective, and accessible therapeutic alternatives that minimize adverse effects while maintaining efficacy.

Phytoconstituents derived from medicinal plants present a promising alternative to conventional psoriasis treatments due to their potent anti-inflammatory, immunomodulatory, and antioxidant properties. Several bioactive compounds, including curcumin and silybin, have demonstrated efficacy in modulating multiple inflammatory pathways, suggesting their potential as multi-target therapeutic agents [14]. Medicinal plants such as *Alstonia scholaris* and *Wrightia tinctoria*, traditionally used in dermatological disorders, have shown significant anti-psoriatic activity by inhibiting keratinocyte proliferation, inducing apoptosis, and reducing inflammatory mediators like IL-8 and RANTES [15]. Other plants, including *Betula utilis* and *Tribulus terrestris*, have also been explored for their anti-psoriatic potential, with studies highlighting their effectiveness in in silico and in vivo models [16,17]. Additionally, isoflavones from soybeans have demonstrated potential for topical application in reducing psoriatic lesions, further supporting the role of plant-derived compounds in psoriasis management [18]. These findings emphasize the potential of phytochemicals as safer, multi-targeted therapeutic agents, reinforcing their role in the development of psoriasis treatment strategies.

In our study, *Alstonia scholaris* and *Wrightia tinctoria* were chosen due to their promising phytochemical profiles. *Alstonia scholaris* contains many bioactive compounds like Alstolenine, which show strong affinity for psoriasis-related targets such as the TRPV3 ion channel and exhibit anti-inflammatory and wound-healing properties [15,19]. Similarly, *Wrightia tinctoria*, traditionally used in Siddha medicine, contains compounds such as lupeol and stigmasterol, which are known for their anti-inflammatory and skin-protective effects [14,20,21,22].

Building upon this, we aimed to identify chloroform-based fractions of *Alstonia scholaris* and *Wrightia tinctoria* extracts with potent anti-psoriatic activity from these plants by isolating and evaluating their bioactive components. Unlike crude extracts, fractionation allows for the enrichment of specific phytoconstituents with enhanced anti-inflammatory, immunomodulatory, and anti-proliferative properties, while minimizing cytotoxic effects. Through a combination of in vitro studies using HaCaT keratinocytes and in vivo experiments on psoriasis-induced mouse models, we provide comprehensive evidence of these fractions inhibiting keratinocyte proliferation, inducing apoptosis, and modulating key inflammatory mediators. By integrating traditional medicinal knowledge with modern pharmacological validation, our study offers a targeted approach to identifying potent bioactive molecules, paving the way for safer, more effective, and accessible plant-based therapies for psoriasis.

## 2. Results

### 2.1. Gas Chromatography-Mass Spectrometry (GC-MS) Analysis and Chemical Characterization of the Crude Extracts

GC–MS analysis of the crude extract of *Alstonia scholaris* revealed the presence of several bioactive compounds with retention times (RT) ranging from 12.43 to 15.11 min (Table 1). At RT 12.43 min, oxirane, tetradecanal, pentadecanal, vinyl lauryl ether, and dodecanal were identified. At RT 13.48 min, compounds such as 1-ethoxypentan-3-ol, hexanol, and rhamnitol were detected. The analysis also identified aziridine and quinolinedione at RT 14.94 min. Finally, compounds including 1,3-dioxane-2-propanol, carboxylic acid, and dimethyldiaziridine were detected at RT 15.11 min.

**Figure 1:**
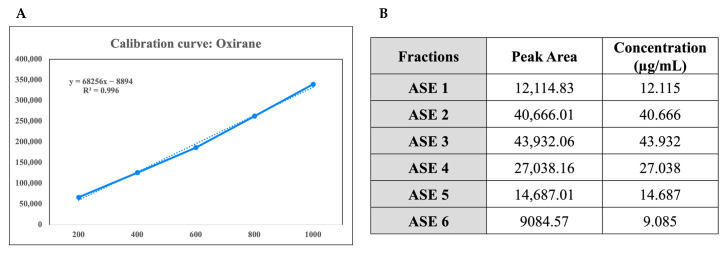
(**A**) Calibration curve of oxirane (marker compound) concentrations ranging from 200 to 1000 ppm. The plot shows a linear correlation (R^2^ = 0.996) between oxirane concentration and peak area, as determined by chromatographic analysis. The linear regression equation is y = mx + c, where y represents the peak area and x represents the oxirane concentration in ppm. This calibration curve was used to quantify oxirane content in fractions ASE 1–ASE 6 of *A. scholaris*. (**B**) Quantification of oxirane content in fractions (ASE 1–ASE 6) of *A. scholaris* using chromatographic analysis. The oxirane content in each fraction was calculated based on a standard calibration curve (**A**) established with oxirane concentrations ranging from 200 to 1000 ppm. Data are represented as mean ± SD of three independent measurements.

To assess the chemical composition of fractions of *A. scholaris*, oxirane was used as a marker compound due to its significant presence and consistent detectability in the crude extract, as revealed by GC–MS analysis. Chromatographic analysis was conducted to quantify oxirane content in the fractions using a standard calibration curve (Figure 1A). The calibration curve, established with oxirane concentrations ranging from 200 to 1000 ppm, exhibited a strong linear correlation between concentration and peak area, with areas increasing from 66,027.5 at 200 ppm to 339,117.37 at 1000 ppm. Using this calibration curve, the oxirane content in six fractions (ASE 1–ASE 6) was quantified (Figure 1B).

Similarly, GC-MS analysis of ethanolic extract of *Wrightia tinctoria* identified multiple compounds at distinct RTs (Table 2). At 12:48 min, compounds including *N*-[4-Aminobutyl] aziridine and 5-Aziridinopentanol were detected, alongside Methyldicyanophosphine and Tris(aziridinomethyl)hydrazine. At 13:44 min, compounds such as 4(1H)-Pyridinone, 2,3-dihydro-1-methyl, Tropinone, 6β-methoxy-, (+), and Pyrazol-4-amine were identified. At 14:86 min, the compounds identified included (E)-Tetradec-2-enal, 3,7,11-Trimethyldodecylacetate, Hexadecane, 1-(ethenyloxy), and 1-Eicosanol.

**Figure 2:**
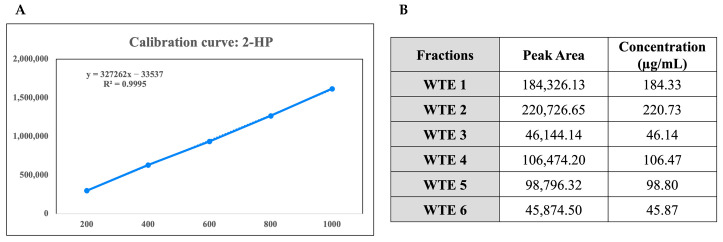
(**A**) Calibration curve of 2-hydroxypyridine (2-HP, marker compound) concentrations ranging from 200 to 1000 ppm. The plot shows a linear correlation (R^2^ = 0.9995) between 2-hydroxypyridine concentration and peak area, as determined by chromatographic analysis. The linear regression equation is *y = mx + c*, where *y* represents the peak area and *x* represents the 2-hydroxypyridine concentration in ppm. This calibration curve was used to quantify 2-hydroxypyridine content in fractions of *W. tinctoria*. (**B**) Quantification of 2-hydroxypyridine content in fractions of *W. tinctoria* using chromatographic analysis. The 2-hydroxypyridine content in each fraction was calculated based on a standard calibration curve (**A**) established with 2-hydroxypyridine concentrations ranging from 200 to 1000 ppm. Data are represented as mean ± SD of three independent measurements.

To evaluate the chemical composition of the fractions of *Wrightia tinctoria*, 2-hydroxypyridine (2-HP) was selected as the marker compound due to its prominent presence and consistent detectability in the crude extract, as demonstrated by GC–MS analysis. Chromatographic analysis was performed to quantify the 2-hydroxypyridine content in the fractions using a standard calibration curve (Figure 2A). The calibration curve was established with 2-hydroxypyridine concentrations ranging from 200 to 1000 ppm, showing a strong linear correlation between concentration and peak area, with areas increasing from 297,370.57 at 200 ppm to 1,615,369.71 at 1000 ppm. The 2-HP content in six fractions (WTE 1–WTE 6) was determined based on this calibration (Figure 2B).

### 2.2. In Vitro Experiment Results

#### 2.2.1. ASE and WTE Fractions Show Anti-Proliferative Effects on HaCaT Cells

All six fractions of the ethanolic extracts of both *A. scholaris* (ASE) and *W. tinctoria* (WTE) were evaluated for their anti-proliferative potential on HaCaT cells using the MTT assay at concentrations ranging from 0.1 μg/mL to 200 μg/mL in HaCaT cells over 24, 48, and 72 h. Results were normalized against untreated control cells and curcumin (positive control), which exhibited a dose-dependent increase in proliferation inhibition and achieved over 60% anti-proliferative activity at a 50 µM dose.

**Figure 3:**
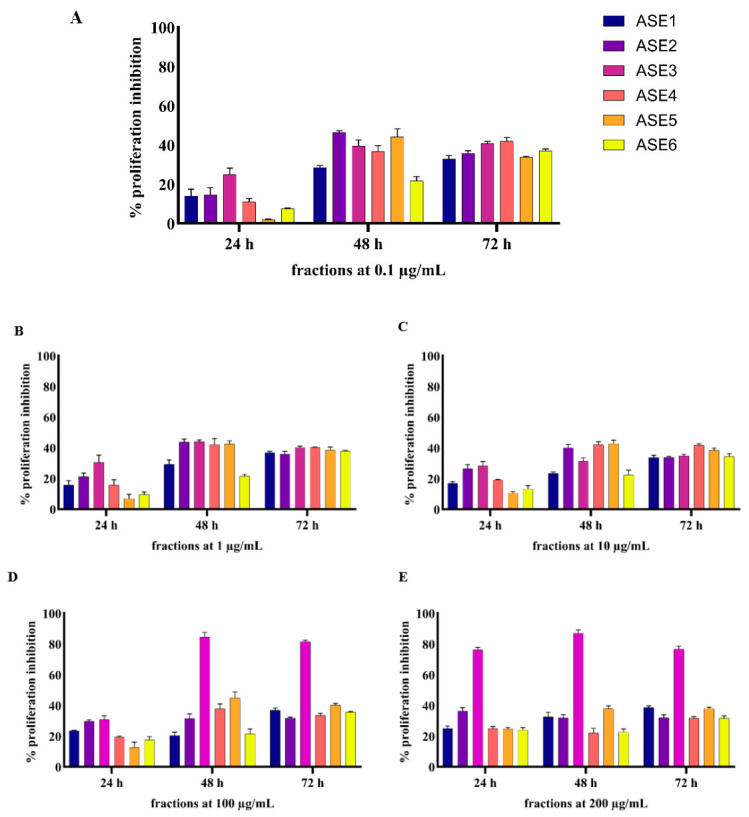
Anti-proliferative effects of ASE1, ASE2, ASE3, ASE4, ASE5, and ASE6 on HaCaT cells. HaCaT cells (10,000 cells/well) were treated with varying concentrations of each fraction (**A**) 0.1 µg/mL, (**B**) 1 µg/mL, (**C**) 10 µg/mL, (**D**) 100 µg/mL, and (**E**) 200 µg/mL for 24, 48, and 72 h. Cell proliferation was assessed using the MTT assay. Curcumin was used as the positive control, and DMSO-treated cells served as the vehicle control. Data are normalized and presented as Mean ± S.E.M. from three independent experiments (n = 3).

**Figure 4:**
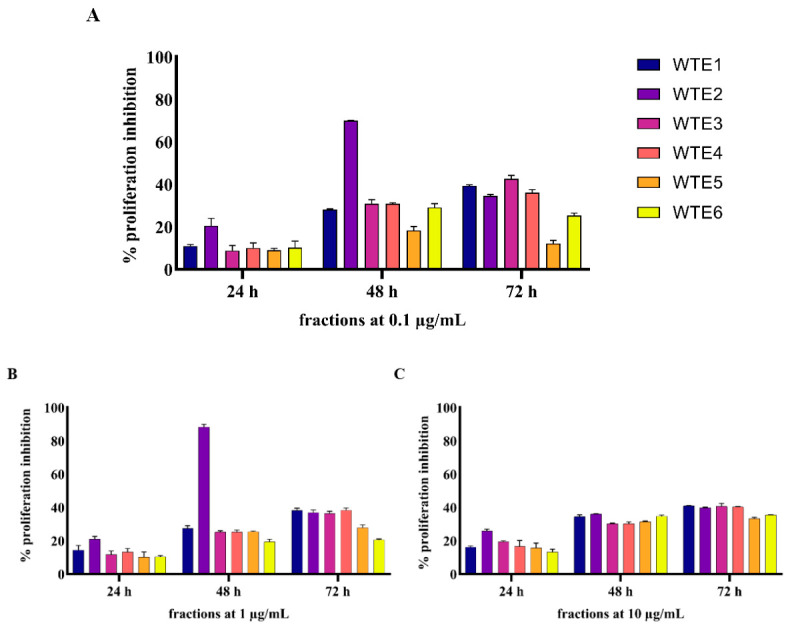

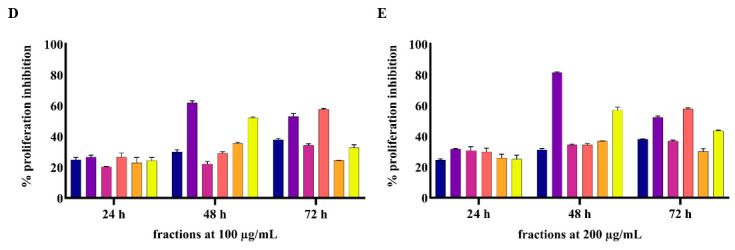
Anti-proliferative effects of WTE1, WTE2, WTE3, WTE4, WTE5, and WTE6 on HaCaT cells. HaCaT cells (10,000 cells/well) were treated with varying concentrations of each fraction (**A**) 0.1 µg/mL, (**B**) 1 µg/mL, (**C**) 10 µg/mL, (**D**) 100 µg/mL, and (**E**) 200 µg/mL for 24, 48, and 72 h. Cell proliferation was assessed using the MTT assay. Curcumin was used as the positive control and DMSO-treated cells served as the vehicle control. Data are normalized and presented as Mean ± S.E.M. from three independent experiments (n = 3).

The MTT assay results indicated that both *A. scholaris* and *W. tinctoria* extracts show significant anti-proliferative effects on HaCaT cells, with inhibition levels varying depending on fraction concentration and treatment time (Figure 3 and Figure 4). All fractions showed significant inhibition of cell proliferation at the 48 h and 72 h treatment points.

Specifically, ASE3 emerged as the most potent fraction from *A. scholaris*, displaying a strong, dose-dependent, and sustained inhibition of cell proliferation, with a nearly 25% inhibition rate at 0.1 µg/mL and reaching 76.28% at 200 µg/mL after 24 h. At 48 h, ASE3 achieved a peak inhibition of 87.1% at 200 µg/mL, which remained high at 72 h with 76.72% inhibition at the same concentration, highlighting its strong and sustained effect. ASE2 also demonstrated considerable anti-proliferative activity, particularly at lower concentrations, with a maximum inhibition of 46.3% at 0.1 µg/mL after 48 h, making it the second most viable fraction. In contrast, all other ASE fractions displayed lower anti-proliferative activity (Figure 3).

Among the *W. tinctoria* fractions, WTE2 showed significant anti-proliferative activity, with inhibition starting at 20% for a 0.1 µg/mL dose after 24 h and increasing to over 69% after 48 h. WTE2 reached a maximum inhibition of 88.3% at a 1 µg/mL post 48 h treatment and maintained substantial inhibition at 72 h, with over 50% inhibition at 100 µg/mL and 200 µg/mL concentrations. Furthermore, WTE3 and WTE4 exhibited significant inhibition levels across all doses, peaking at 42.5% at 0.1 µg/mL and over 57% at 100–200 µg/mL, respectively, at 72 h. WTE1 and WTE5 showed lower inhibition (<40%), while WTE6 showed a notable anti-proliferative activity of 57% at 200 µg/mL after 48 h (Figure 4).

Overall, for both *A. scholaris* and *W. tinctoria* extracts, proliferation inhibition increased significantly for all fractions at higher concentrations irrespective of treatment duration. ASE3 exhibited the highest anti-proliferative activity at higher concentrations, while WTE2 demonstrated substantial inhibition at lower concentrations, particularly at the 48 h time-point.

#### 2.2.2. ASE and WTE Fractions Induced Activation of Apoptotic Response

Psoriasis is characterized by the hyperproliferation of keratinocytes and impaired apoptosis, making the activation of apoptosis a critical therapeutic target. Therefore, we assessed the potential of ASE and WTE fractions to induce apoptosis in HaCaT cells, using membrane depolarization assays.

Mitochondrial membrane potential (MMP), Δψ_M_, is one of the key indicators of cell health and commonly determined by JC-1 assay reference. The impact of ASE and WTE fractions to induce apoptosis in HaCaT cells was assessed by exposing treated cells to JC-1 dye. The Δψ_M_ was monitored by flow cytometry, detecting red fluorescent J-aggregates and green fluorescent J-monomers, followed by calculating the membrane depolarization (reduction in MMP) for each treatment. In contrast to untreated control cells, the treatment with each fraction resulted in an increased apoptotic population (depolarized) with varying response across all concentrations (0.1 µg/mL, 1 µg/mL, 10 µg/mL, 100 µg/mL and 200 µg/mL).

**Figure 5:**
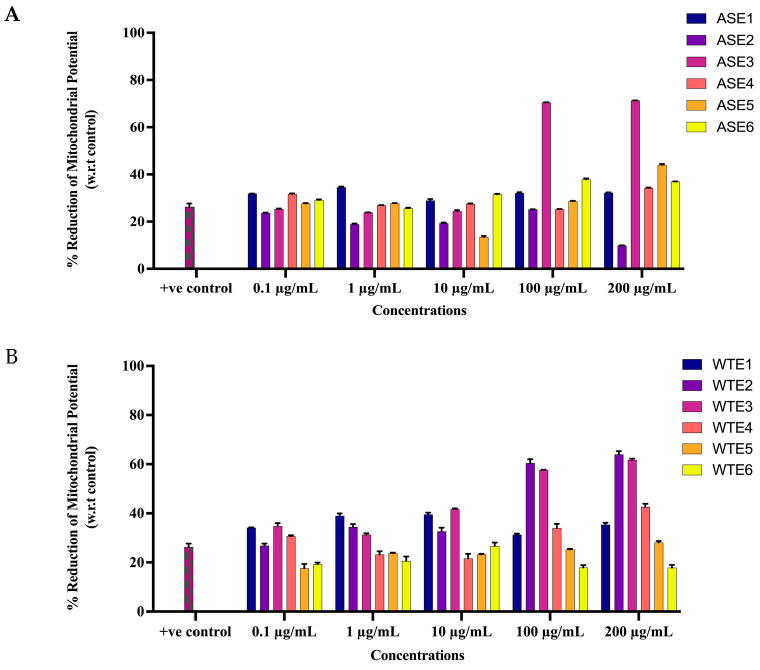
Apoptotic effects of ASE and WTE fractions on HaCaT cells. HaCaT cells (10,000 cells/well) were treated with ASE fractions (**A**) and WTE fractions (**B**), and apoptosis was measured using the JC-1 assay. Curcumin was used as the positive control. Data are normalized and presented as Mean ± S.E.M. from three independent experiments (n = 3).

Significant membrane depolarization was observed in cells treated with higher concentrations of ASE2, ASE3, WTE2, and WTE3. ASE3 showed a dose-dependent increase in membrane depolarization, with a significant MMP reduction of 70.39% and 71.37% in over 60% cells at 100 and 200 µg/mL, respectively, whereas ASE2 was the second most viable fraction in reducing MMP with 28.85% and 44.00% at 100 and 200 µg/mL, respectively (Figure 5A). WTE2 exhibited a strong dose-dependent increase in membrane depolarization, particularly at 100 and 200 µg/mL with 60.44% and 63.95%, respectively. Similarly, WTE3 demonstrated a significant MMP reduction of over 50% at 100 and 200 µg/mL (Figure 5B).

#### 2.2.3. ASE and WTE Fractions Inhibited the Secretion of Inflammatory Mediator IL-8

**Figure 6:**
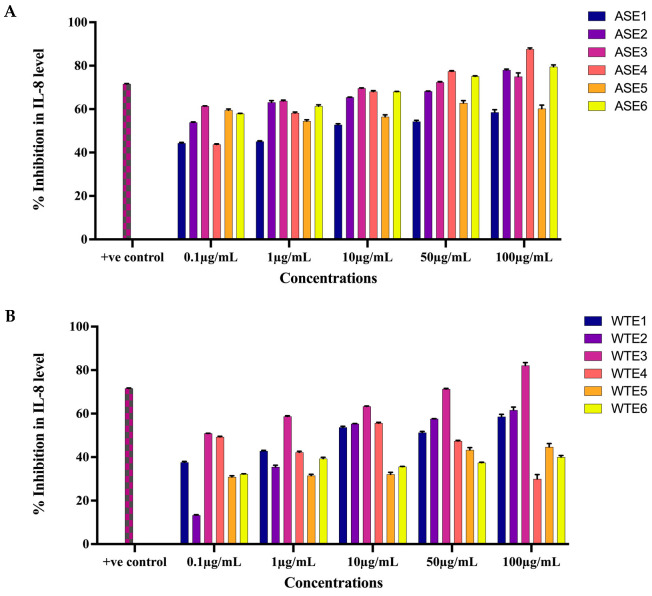
Inhibition of IL-8 levels in TNF-α-induced HaCaT cells by ASE and WTE fractions. HaCaT cells (10,000 cells/well) were treated with ASE fractions (**A**) and WTE fractions (**B**) to assess IL-8 levels. Methotrexate was used as the positive control. Data are normalized and presented as Mean ± S.E.M. from three independent experiments (n = 3).

The effects of ASE and WTE fractions on the secretion of key pro-inflammatory markers were assessed in HaCaT cells treated with TNF-α (10 ng/mL) over a 24 h period, using concentrations ranging from 0.1 μg/mL to 100 μg/mL. All fractions, except for ASE5, WTE4, and WTE6, exhibited a significant dose-dependent inhibition of IL-8 secretion. Whereas ASE2 inhibited more than 85% of IL-8 levels at 100 µg/mL, ASE3, the second most viable fraction, inhibited 72.4% IL-8 levels at 50 µg/mL and 75.0% at 100 µg/mL (Figure 6A). WTE3 exhibited more than 82% inhibition in IL-8 levels at 100 µg/mL. Similarly, WTE2 demonstrated a substantial reduction in IL-8 secretion of more than 55% at concentrations 10 µg/mL, 50 µg/mL, and 100 µg/mL (Figure 6B). Therefore, ASE2, ASE3, WTE2, and WTE3 are most viable fractions in inhibiting the IL-8 levels.

#### 2.2.4. ASE and WTE Fractions Inhibited the Secretion of Inflammatory Mediator RANTES

**Figure 7:**
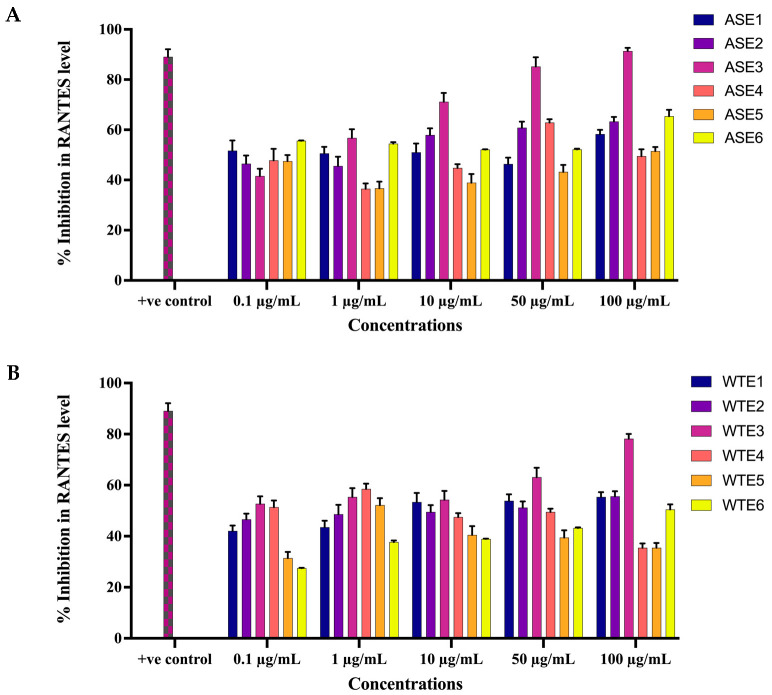
Inhibition of RANTES levels in TNF-α-induced HaCaT cells by ASE and WTE fractions. HaCaT cells (10,000 cells/well) were treated with ASE fractions (**A**) and WTE fractions (**B**) to assess RANTES levels. Methotrexate was used as the positive control. Data are normalized and presented as Mean ± S.E.M. from three independent experiments (n = 3).

The ability of ASE and WTE fractions to downregulate the secretion of RANTES from HaCaT cells was assessed at concentrations of 0.1 μg/mL and 100 μg/mL post-24 h treatment. Treatment with ASE1 at all concentrations, except 50 μg/mL, resulted in more than 50% inhibition of RANTES levels (Figure 7A). Specifically, ASE2 (0.1 μg/mL and 100 μg/mL) and ASE3 (0.1 μg/mL and 100 μg/mL) showed a dose-dependent reduction in RANTES secretion, with reductions ranging from 46.5% to 63.3% and 41.6% to 91.4%, respectively, against TNF-α stimulated cells. However, ASE4, ASE5, and ASE6 did not demonstrate a dose-dependent reduction (Figure 7A).

For the WTE fractions, treatment with WTE1 (0.1 μg/mL and 100 μg/mL), WTE2 (0.1 μg/mL and 100 μg/mL), and WTE3 (0.1 μg/mL and 100 μg/mL) resulted in a dose-dependent reduction in RANTES secretion by 42.1% to 55.4%, 46.6% to 55.6%, and 52.7% to 78.2%, respectively, compared to TNF-α stimulated cells (Figure 7B). Conversely, treatment with WTE4, WTE5, and WTE6 showed maximum inhibition of RANTES secretion at concentrations of 1 μg/mL, 1 μg/mL, and 100 μg/mL, with inhibitions of 58.5%, 52.2%, and 50.5%, respectively, compared to TNF-α stimulated cells (Figure 7B).

### 2.3. In Vivo Experiment Results

#### 2.3.1. Measurement of Body Weight and Skin Edema

**Figure 8:**
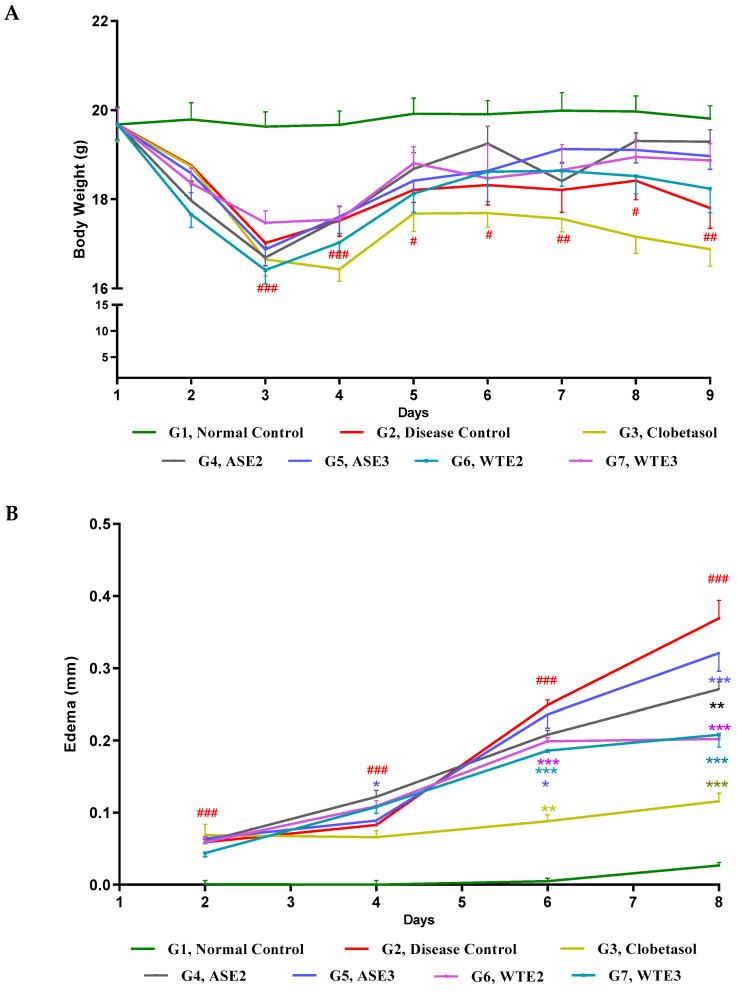
Evaluation of daily body weight (**A**) and skin edema (**B**) post-treatment with the positive control, ASE2, ASE3, WTE2, and WTE3 on an imiquimod (IMQ)-induced psoriasis-like mouse model. Clobetasol was used as the positive control. Data were statistically analyzed by two-way ANOVA followed by Bonferroni’s test. Statistical significance is indicated as follows: comparison between the disease control group (G2) and the normal control group (G1): *p* < 0.05 (#), *p* < 0.01 (##), *p* < 0.001 (###). Comparison between treatment groups (G3–G7) and the disease control group (G2): *p* < 0.05 (*), *p* < 0.01 (**), and *p* < 0.001 (***).

To examine the anti-psoriatic potential of selected ASE and WTE fractions in vivo, we first evaluated the impact of these active fractions on body weight and skin edema on the shaved dorsal back of IMQ-induced psoriatic mice. Body weight and ear thickness were recorded daily before IMQ-induction. The untreated control group maintained a stable body weight of approximately 19.5 g throughout the study period. In contrast, a positive control group treated with clobetasol showed a significant reduction in body weight by 2.78 g, with noticeable changes as early as day 3 of the 8-day induction and treatment period. A similar pattern of weight reduction was observed in mice treated with ASE2, ASE3, WTE2, and WTE3. However, towards the end of the study, mice treated with these fractions began to regain body weight, reaching 18–19 g, indicating a recovery trend. Particularly, mice in the clobetasol and disease control groups did not regain their body weight, which remained below 17–18 g (Figure 8A).

The decrease in body weight until day 3 could be attributed to the initial systemic response to the IMQ treatment, which often induces stress, reduced food intake, and metabolic changes in mice, leading to temporary weight loss. Additionally, inflammation and immune activation can contribute to a catabolic state, further affecting body weight.

Furthermore, regarding the randomized body weight trend in G4 at day 7, it is possible that individual variability in response to treatment, differences in recovery rates, or compensatory feeding behavior led to fluctuations. The treatment might have influenced metabolic adaptation differently among mice, leading to inconsistent weight regain patterns.

Skin edema, indicated by changes in ear thickness, was measured every alternate day of treatment, starting on day 2. The disease control group exhibited a linear increase in ear thickness, reaching 0.5 mm by day 8. In contrast, on day 6 and day 8, the clobetasol-treated group consistently maintained edema at nearly 0.1 mm throughout the study. Mice treated with ASE2, ASE3, WTE2, and WTE3 displayed a controlled level of edema by days 6–8, with measurements ranging between 0.18 mm and 0.3 mm. This suggests that these fractions were effective in managing skin edema induced by IMQ, although not as effectively as clobetasol, but significantly better when compared to the disease control (Figure 8B).

#### 2.3.2. Splenomegaly Assessment

**Figure 9:**
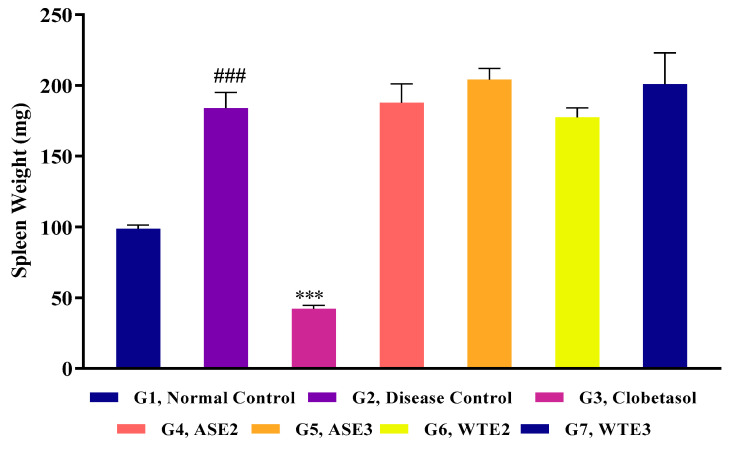
Evaluation of spleen weight post-treatment with the positive control, ASE2, ASE3, WTE2, and WTE3 on imiquimod-induced psoriasis-like mouse model. Clobetasol was used as the positive control. Data were statistically analyzed by two-way ANOVA followed by Bonferroni’s test. Statistical significance is indicated as follows: comparison between the disease control group (G2) and the normal control group (G1): *p* < 0.001 (###). Comparison between treatment groups (G3–G7) and the disease control group (G2): *p* < 0.001 (***).

Following the 8-day treatment period, the impact of ASE and WTE fractions on spleen weight was evaluated to understand their potential systemic effects and immune modulation properties. The results show that the spleen weight of the disease control group was considerably greater than that of the control group. However, the clobetasol-treated group presented a significant reduction in spleen weight, indicating its effectiveness in reducing systemic inflammation. However, treatments with ASE2, ASE3, WTE2, and WTE3 did not result in a reduction in spleen weight (Figure 9). The spleen weights in these groups remained comparable to those observed in the disease control group, suggesting that while the fractions were effective in managing local symptoms such as skin edema, they did not exert a similar systemic anti-inflammatory effect as clobetasol.

#### 2.3.3. Assessment of PASI Score Post-Treatment

The evaluation of the PASI score post-treatment for each active fraction was conducted to determine their efficacy in managing psoriasis symptoms. The vehicle-treated group (G1) did not exhibit any signs of inflammation, serving as the normal control. Starting from day 4, the disease control group (G2) demonstrated a significant increase in cumulative PASI scores, confirming the successful induction of psoriasis.

**Figure 10:**
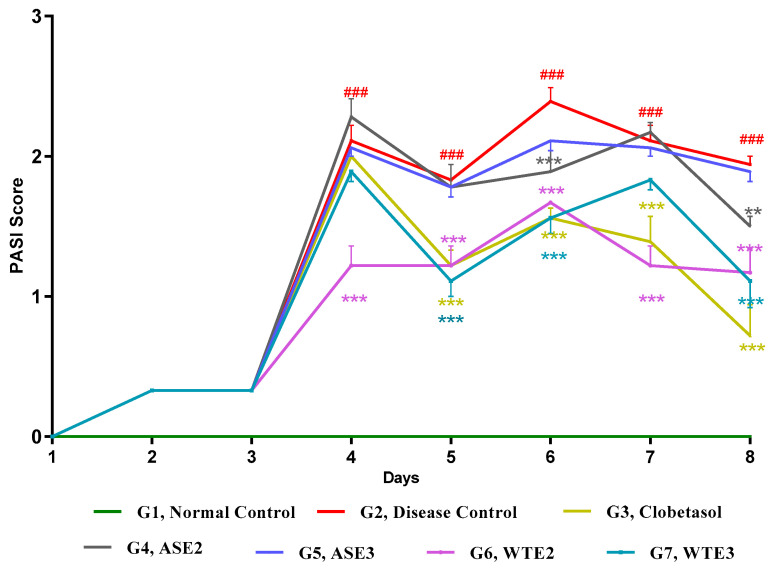
Assessment of PASI scores following treatment with the positive control, ASE2, ASE3, WTE2, and WTE3 in an imiquimod-induced psoriasis-like mouse model. Clobetasol was used as the positive control. Data were statistically analyzed by two-way ANOVA followed by Bonferroni’s test. Statistical significance is indicated as follows: comparison between the disease control group (G2) and the normal control group (G1): *p* < 0.001 (###). Comparison between treatment groups (G3–G7) and the disease control group (G2): *p* < 0.01 (**) and *p* < 0.001 (***).

In the clobetasol-treated group (G3), a significant reduction in PASI scores was observed from day 5 onwards compared to the disease control group (G2), highlighting the efficacy of clobetasol in mitigating psoriatic symptoms. Among the fractions, the ASE2 treated group (G4) showed a significant reduction in PASI scores on days 6 and 8 compared to the disease control group (G2), indicating some efficacy in reducing psoriatic symptoms. However, ASE3 (G5) did not exhibit a significant change in PASI scores, suggesting its limited efficacy (Figure 10).

On the other hand, WTE2 and WTE3 treatments resulted in much-reduced PASI scores. WTE2 showed a significant reduction from day 4 onwards, and WTE3 showed a comparable reduction starting from day 5, both of which were comparable to the clobetasol-treated group. This indicates that, although less pronounced in ASE2 and ASE3, the WTE2 and WTE3 fractions were efficacious in reducing psoriatic symptoms, similar to the standard clobetasol treatment (Figure 10).

#### 2.3.4. Assessment of Ear Punch Biopsy Weight Post-Treatment

**Figure 11:**
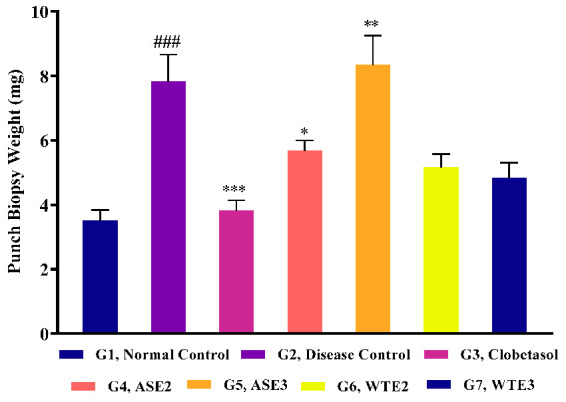
Effect of the positive control, ASE2, ASE3, WTE2, and WTE3 on ear punch biopsy weight in imiquimod-induced psoriasis-like mouse model. Clobetasol was used as the positive control. Data were statistically analyzed by two-way ANOVA followed by Bonferroni’s test. Statistical significance is indicated as follows: comparison between the disease control group (G2) and the normal control group (G1): *p* < 0.001 (###). Comparison between treatment groups (G3–G7) and the disease control group (G2): *p* < 0.05 (*), *p* < 0.01 (**) and *p* < 0.001 (***).

Ear punch biopsy weights were measured post-treatment to evaluate the extent of inflammation and hyperplasia in the IMQ-induced psoriatic mice. On day 9, all experimental animals were euthanized, and a standard 4 mm ear punch biopsy was collected and weighed. The normal control group exhibited an ear punch biopsy weight of 3.50 ± 0.34 mg, serving as the baseline for comparison. The disease control group (G2) showed a significantly increased biopsy weight (7.83 ± 0.83 mg), reflecting severe inflammation and hyperplasia due to psoriasis induction (Figure 11).

In the clobetasol-treated group (G3), the biopsy weight was significantly reduced (3.83 ± 0.31 mg), indicating its efficacy in reducing inflammation. Further, ASE2-treated mice (G4) had a biopsy weight of 5.67 ± 0.33 mg, demonstrating moderate efficacy in reducing ear inflammation. The ASE3-treated group (G5), however, showed a biopsy weight (8.33 ± 0.92 mg) that was even higher than the disease control group, suggesting a lack of efficacy in this measure (Figure 11).

WTE2 and WTE3 fractions demonstrated better outcomes, with biopsy weights of 5.17 ± 0.40 mg and 4.83 ± 0.48 mg, respectively. These results indicate that WTE2 and WTE3 were more efficacious in reducing ear inflammation and hyperplasia compared to ASE2 and ASE3, with WTE3 showing anti-inflammatory potency comparable to the clobetasol treatment in our psoriatic model (Figure 11).

#### 2.3.5. Histopathological Evaluation Post-Treatment

**Figure 12:**
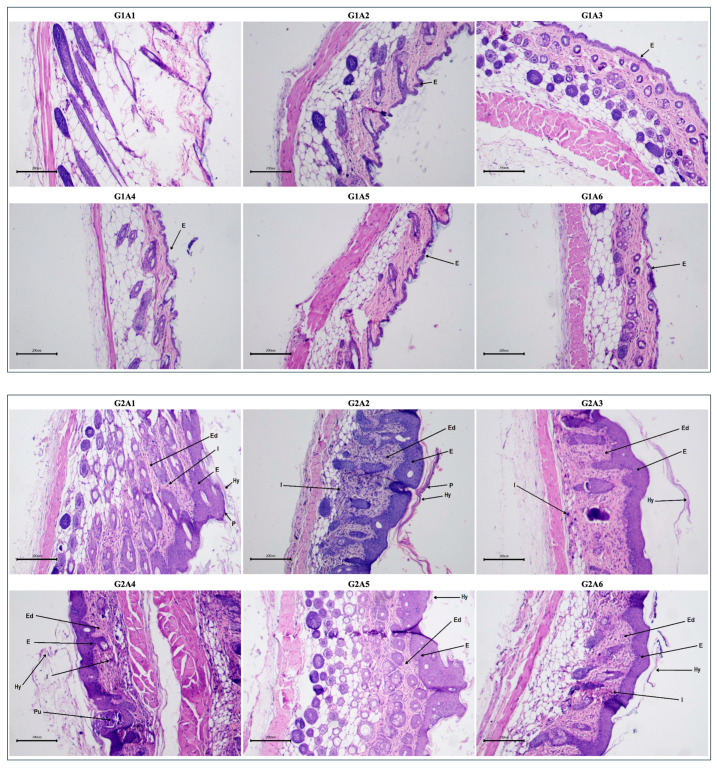

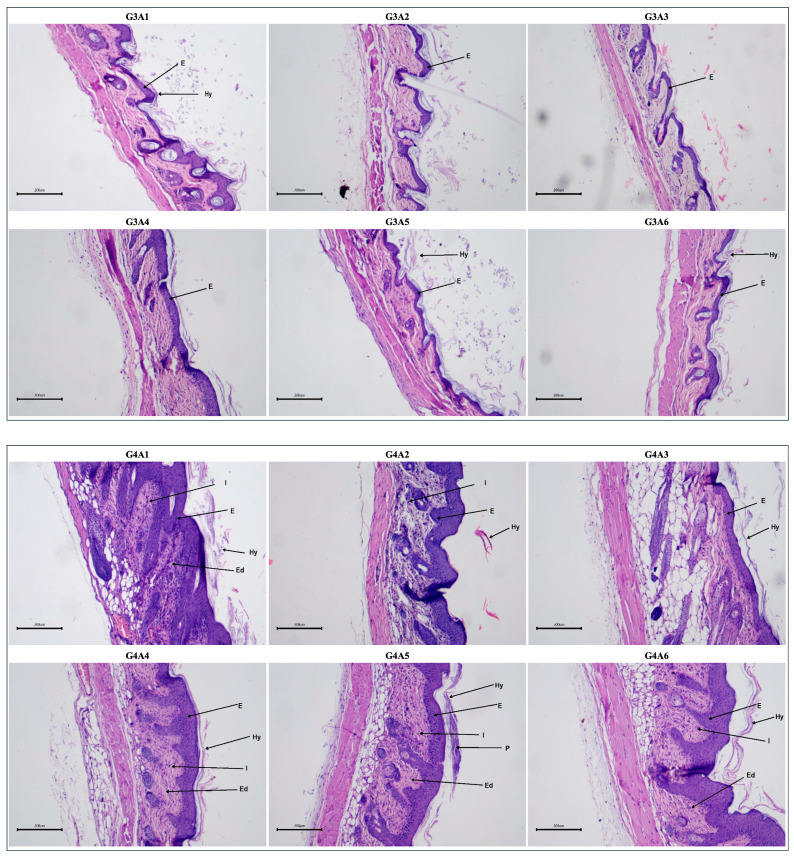

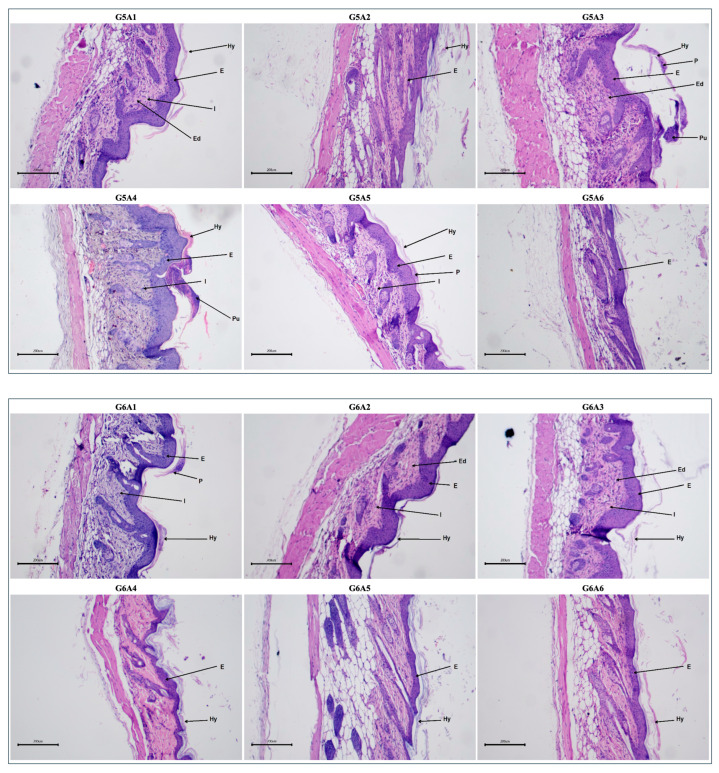

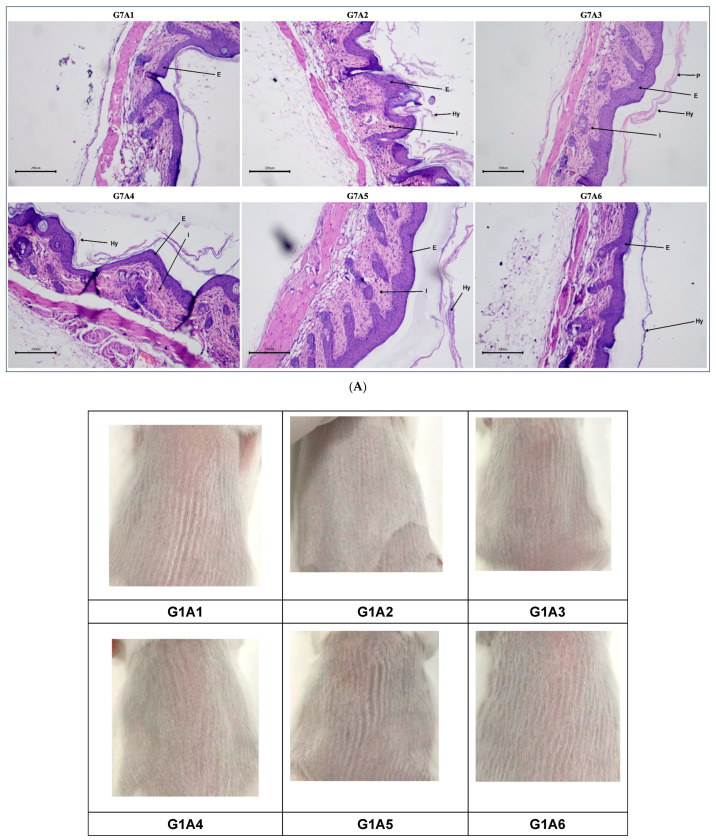

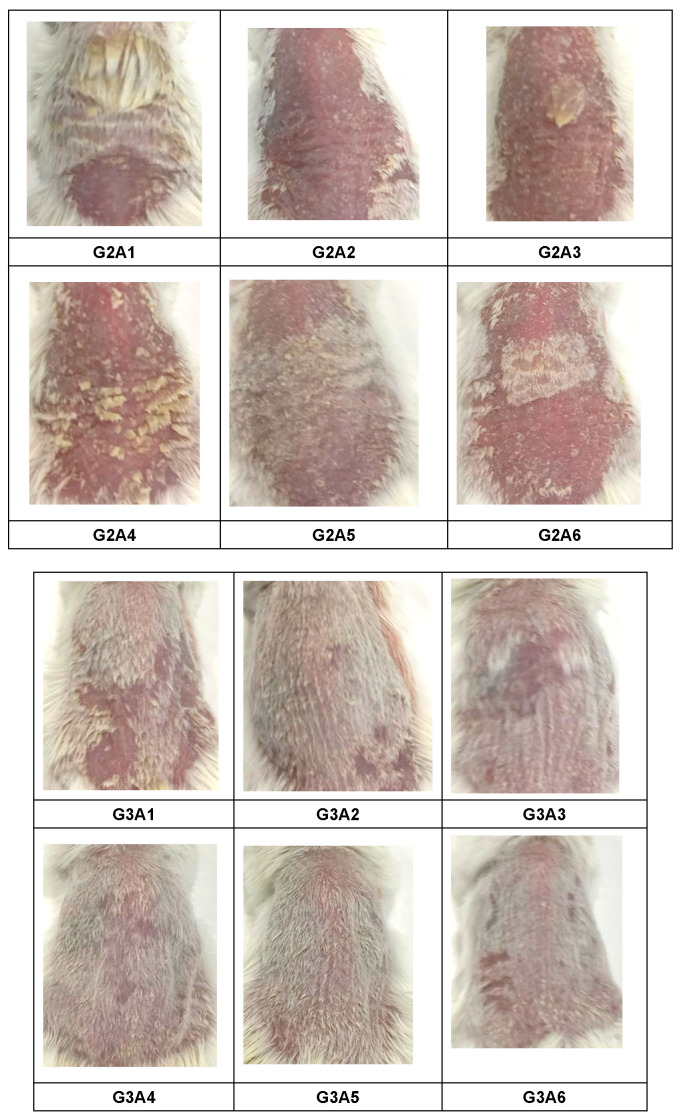

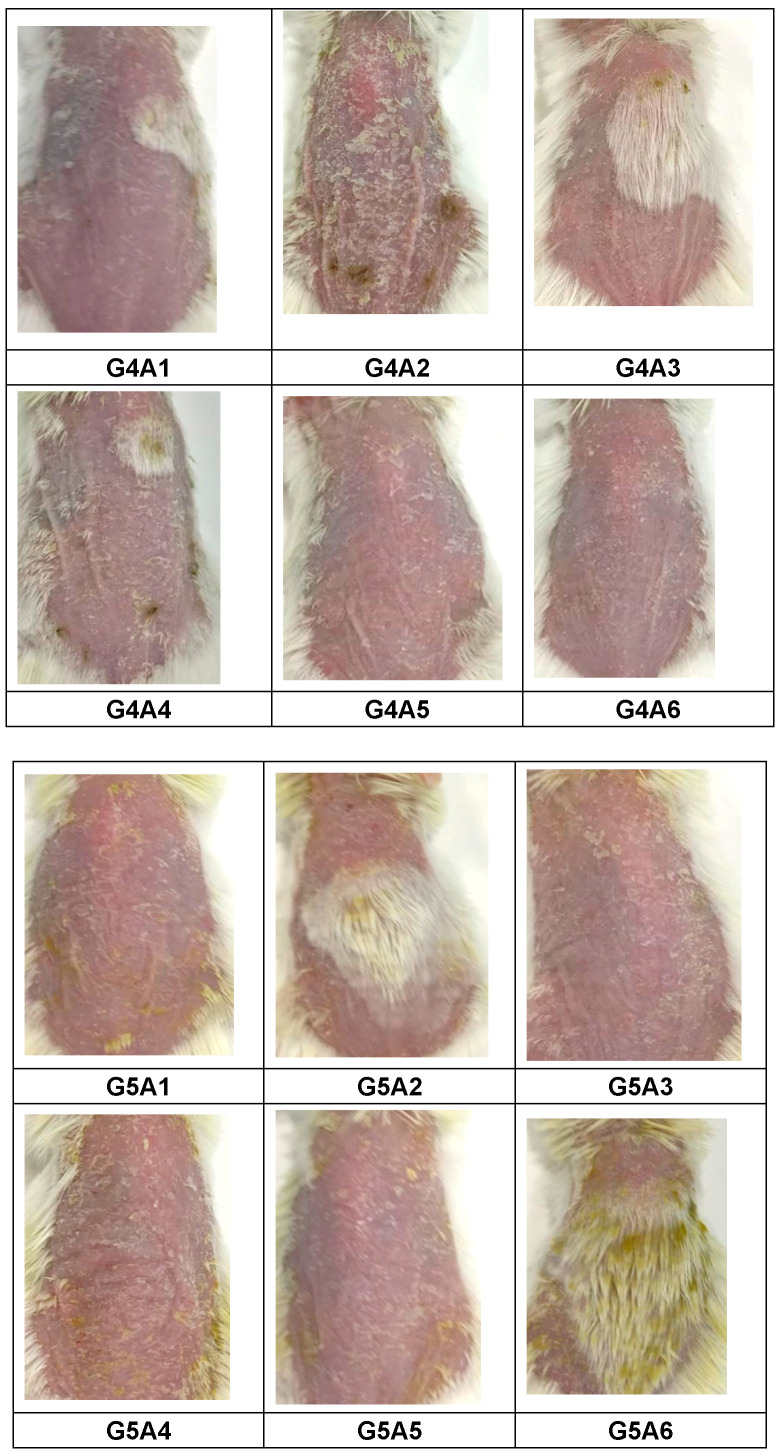

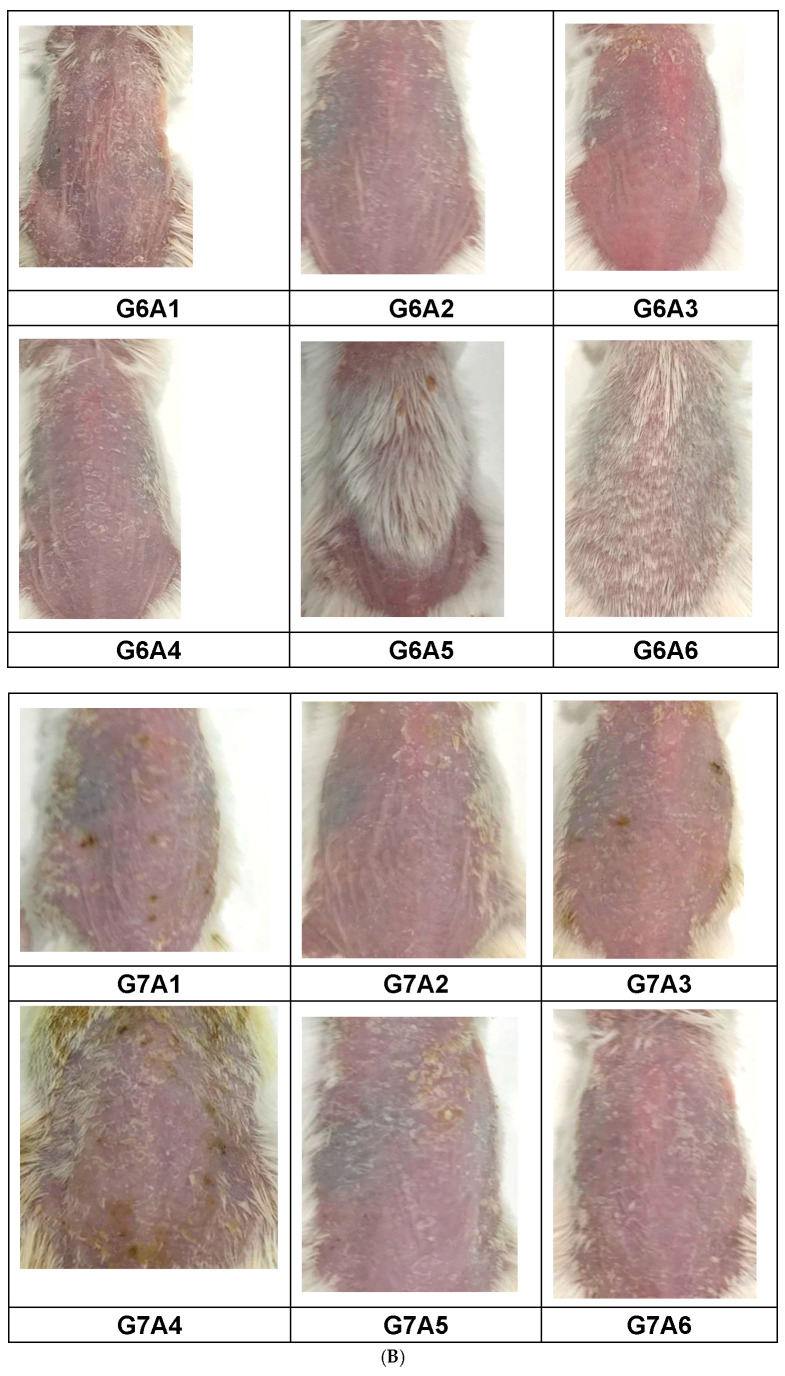
Representative histopathological images of dorsal skin sections (**A**) and macroscopic appearance of the dorsal skin (**B**) from BALB/c mice (n = 6 per group, represented by A1 to A6) following repeated applications of ASE2, ASE3, WTE2, and WTE3. The groups are designed as follows: (**G1**) negative control, (**G2**) disease control, (**G3**) 0.05% clobetasol (positive control), (**G4**) ASE2, (**G5**) ASE3, (**G6**) WTE2, and (**G7**) WTE3. Arrows indicate key histopathological features used to assess disease severity and treatment effects: **E** (epidermal thickening), **Hy** (hyperkeratosis), **P** (parakeratosis), **Pu** (pustule), **I** (inflammatory infiltration), and **Ed** (edema). Scale bar: 200 μm.

Macroscopic assessment of dorsal skin appearance combined with histopathological evaluation of psoriatic skin inflammation involved assessing key parameters such as hyperkeratosis, parakeratosis, epidermal thickness, presence of pustules, edema, and inflammatory cell infiltration Figure 12. Comparative analysis between the disease control group (G2) and treatment groups revealed significant reductions in these parameters, notably in G3 (Clobetasol), G6 (WTE2-treated group), and G7 (WTE3-treated group). Additionally, histopathological scoring indicated that treatment with WTE3 (G7) and WTE2 (G6) substantially ameliorated inflammatory skin symptoms, evidenced by histopathological scores of 22 and 23, respectively. Conversely, histopathological examination of G4 (ASE2-treated group) and G5 (ASE3-treated group) showed lesser reductions in these parameters, resulting in histopathological scores of 34 and 31, respectively.

#### 2.3.6. IL-23 and IL-17 Levels in Serum and Skin Homogenate

The IL-23/IL-17 axis plays a significant role in the pathogenesis of psoriasis [21]. IL-23 promotes the differentiation and proliferation of Th17 cells, which produce IL-17. Elevated levels of these cytokines are associated with the inflammatory response in psoriasis.

**Figure 13:**
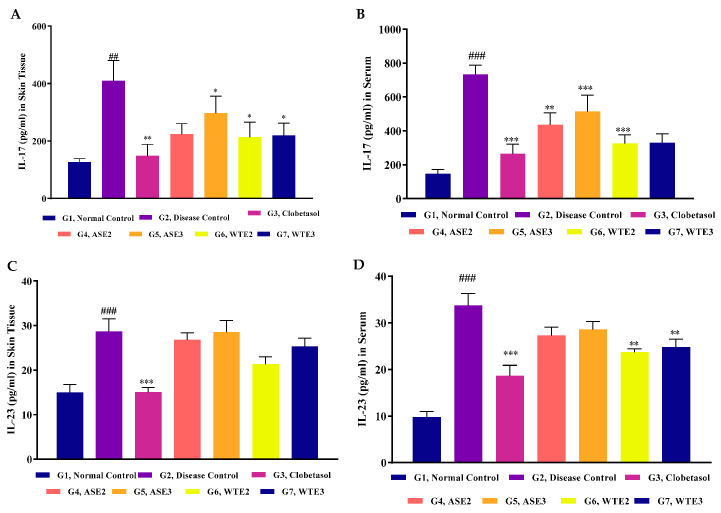
Evaluation of IL-17 in skin homogenate (**A**) and serum (**B**) and IL-23 in skin homogenate (**C**) and serum (**D**) levels post-treatment with positive control, ASE2, ASE3, WTE2, and WTE3 on an imiquimod-induced psoriasis-like mice model. Clobetasol was used as the positive control. Data were statistically analyzed by two-way ANOVA followed by Bonferroni’s test. Statistical significance is indicated as follows: comparison between the disease control group (G2) and the normal control group (G1): *p* < 0.01 (##), *p* < 0.001 (###). Comparison between treatment groups (G3–G7) and the disease control group (G2): *p* < 0.05 (*), *p* < 0.01 (**) and *p* < 0.001 (***).

In the serum, IL-23 levels in the disease control group were over three times higher compared to the untreated control group. Clobetasol treatment effectively reduced IL-23 levels to nearly half of those in the disease control group. While ASE2 and ASE3 treatments resulted in reduced IL-23 levels, these reductions were not statistically significant. In contrast, WTE2 and WTE3 treatments showed significant reductions in IL-23 levels, though they were still higher than those observed in the clobetasol-treated group (Figure 13D). IL-17 levels in the disease control group were approximately five times higher than in the normal control group. Clobetasol treatment brought IL-17 levels back to normal control levels. ASE3 treatment did not significantly reduce IL-17 levels but did achieve a reduction of nearly one-third compared to the disease control group. ASE2 resulted in ~40% IL-17 reductions, whereas WTE3 and WTE2 treatments demonstrated comparable reductions in IL-17 levels to those seen with clobetasol, indicating significant efficacy (Figure 13B).

In the skin homogenate, IL-23 levels in the disease control group were significantly elevated to almost double the levels of the untreated normal control. Clobetasol treatment reduced IL-23 levels back to normal conditions. All four plant extract fractions (ASE2, ASE3, WTE2, and WTE3) showed marginal and non-significant reductions in IL-23 levels compared to the disease control group (Figure 13C). IL-17 levels in the disease control group were significantly increased, more than tripling those of the untreated normal control group. Clobetasol treatment significantly reduced IL-17 levels to normal control levels. While ASE3 treatment resulted in a non-significant reduction in IL-17 by over one-fourth, ASE2, WTE2, and WTE3 treatments significantly reduced IL-17 levels by nearly 50%, demonstrating their potential efficacy in mitigating psoriatic inflammation (Figure 13A).

These results suggest that WTE2 and WTE3 are particularly efficacious in reducing pro-inflammatory cytokine levels both in serum and skin, further supporting their potential as therapeutic agents for psoriasis.

## 3. Discussion

Psoriasis is a chronic, immune-mediated inflammatory skin condition manifested by hyperproliferation of keratinocytes, inflammation, and aberrant immune responses. The pathogenesis of psoriasis involves complex interactions between the immune system and skin cells, particularly the overproduction of pro-inflammatory cytokines such as IL-23 and IL-17 [21,22]. Current therapeutic options for psoriasis include topical corticosteroids, systemic immunosuppressants, and biologics targeting specific cytokines. While these treatments can be effective, they are often associated with significant side effects, limited efficacy, and the potential for relapse upon discontinuation. Consequently, there is a growing interest in identifying novel therapeutic agents with fewer side effects and long-term efficacy. This study aimed to evaluate the anti-psoriatic potential of fractions derived from *Alstonia scholaris* (ASE) and *Wrightia tinctoria* (WTE), two medicinal plants traditionally used for treating skin disorders, by assessing their effects in an IMQ-induced psoriasis-like mouse model.

The GC–MS analysis of crude extracts from *Alstonia scholaris* and *Wrightia tinctoria* identified a diverse range of bioactive compounds that support their therapeutic potential in psoriasis management. In *A. scholaris*, several identified compounds, including oxirane, tetradecanal, pentadecanal, and vinyl lauryl ether, are recognized for their antimicrobial, anti-inflammatory, and antioxidative properties, which are crucial in managing inflammatory skin conditions [23,24,25,26]. Additionally, the detection of 1-ethoxypentan-3-ol, hexanol, and rhamnitol suggests potential roles in skin healing and inflammation control, further reinforcing their relevance for psoriasis treatment [27,28]. The identification of aziridine and quinolinedione at later retention times suggests the presence of nitrogen-containing compounds with potential therapeutic applications in modulating inflammatory pathways [29,30,31].

Similarly, the GC-MS profiling of *W. tinctoria* identified several aziridine derivatives, including *N*-[4-Aminobutyl] aziridine and 5-Aziridinopentanol, which are recognised for their ability to influence oxidative stress and inflammation, significant factors in the pathology of psoriasis [32,33,34]. The identification of compounds such as tropinone and pyrazol-4-amine suggests potential anti-inflammatory and immune-modulatory effects [35]. The compounds (E)-Tetradec-2-enal and 3,7,11-Trimethyldodecylacetate indicate the potential of these extracts in addressing lipid peroxidation, which is associated with skin disorders [36,37]. Salsolidine found in *Wrightia tinctoria* extract is a naturally occurring alkaloid characterized by its isoquinoline structure. It exhibits various pharmacological properties, including antioxidant, anti-inflammatory, and neuroprotective effects, making it a molecule of interest in therapeutic research [38]. These findings highlight the significant bioactivity of *A. scholaris* and *W. tinctoria* extracts, indicating that their chloroform-based fractions may provide notable anti-psoriatic effects through the modulation of inflammatory pathways, oxidative stress, and cellular proliferation, thereby presenting promising opportunities for further therapeutic investigation.

Both *A. scholaris* (AS) and *W. tinctoria* (WT) have long histories of use in traditional medicine for treating various skin conditions and are recognized particularly for their anti-inflammatory, antioxidant, anti-diabetic, anti-cancer, anti-allergic, antipyretic and antimicrobial properties [11,39,40,41]. Both plants exhibit anti-proliferative effects and downregulate pro-inflammatory cytokines, which play a key role in the pathogenesis of psoriasis. Considering these aspects of the phytotherapeutic properties of these plants, we previously reported the anti-psoriatic potential of their crude extracts, demonstrating their efficacy in alleviating psoriatic phenotypes in vitro [15]. Building upon these findings, this study aimed to further explore the therapeutic potential of these plants by fractionating their extracts using a chloroform:methanol gradient system. We then investigated the anti-psoriatic efficacy of these fractions both in vitro using HaCaT cells and in vivo using an IMQ-induced psoriatic mice model. This approach allowed us to identify the specific fractions responsible for the observed anti-psoriatic effects, providing a more detailed understanding of the bioactive components within these plants. Positive controls have been used in experiments to validate the reliability of the experimental system by producing a known and expected response, ensuring the accuracy and reproducibility of results. Curcumin is used as a positive control for proliferation inhibition and apoptosis due to its ability to suppress keratinocyte hyperproliferation and induce apoptosis via NF-κB and MAPK pathway modulation [42,43]. Methotrexate serves as a positive control for inflammatory cytokine inhibition, effectively reducing IL-8 and RANTES levels through its immunosuppressive and anti-inflammatory mechanisms [44]. For in vivo study, clobetasol propionate is used as a positive control due to its potent corticosteroid activity, suppressing epidermal hyperproliferation and inflammatory responses in psoriasis [45].

Psoriasis is characterized by the hyperproliferation of keratinocytes, leading to the formation of thickened, scaly plaques on the skin [46,47,48]. In our study, we observed that all fractions of ASE and WTE effectively inhibited keratinocyte growth in HaCaT cell cultures, suggesting their potential anti-psoriatic activity. Interestingly, the inhibition was more pronounced at 72 h compared to 48 h, indicating a time-dependent effect. This decline in proliferation can be attributed to multiple factors, including the cytotoxic effects of the fractions and the onset of contact inhibition due to over-confluency.

At 48 h, the active fractions exhibited significant anti-proliferative effects, likely mediated by apoptosis induction or cell cycle arrest, mechanisms well-documented in studies on plant-derived compounds that exert cytotoxicity through mitochondrial dysfunction and caspase activation [49,50]. However, by 72 h, additional factors such as contact inhibition became prominent. This well-documented phenomenon occurs when cells reach confluency, leading to growth arrest due to cell–cell interactions and spatial limitations [51]. Furthermore, prolonged culture without passaging results in nutrient depletion, metabolic waste accumulation, and detachment of non-viable cells, all of which contribute to reduced proliferation [52]. Therefore, while the cytotoxic properties of the fractions play a significant role, the impact of over-confluency and subsequent cell detachment cannot be overlooked.

A particularly intriguing trend was observed with WTE2, where proliferation initially declined at 10 µg/mL before resuming a dose-dependent inhibition from 10 to 200 µg/mL. This non-linear response underscores the complexity of plant fractions, which contain multiple bioactive compounds that can interact in synergistic or antagonistic ways [53]. At lower concentrations, certain constituents within WTE2 may exert protective or proliferative effects by activating survival pathways or mild stress responses that enhance cell viability [54]. However, at 10 µg/mL, a shift in the dominant bioactive components may transiently induce cytostatic or inhibitory effects, potentially through oxidative stress or mitochondrial dysfunction, leading to a temporary decline in proliferation [55]. As the concentration increases, cytotoxic constituents likely become more dominant, reinforcing apoptosis and cell cycle arrest, resulting in a clear dose-dependent reduction in proliferation [56].

These findings highlight the dynamic and complex nature of plant fractions, which do not behave as single, linear-acting molecules but rather as intricate mixtures with concentration-dependent biological effects. Understanding these interactions is essential for optimizing their therapeutic potential in psoriasis treatment.

Moreover, four fractions from both the plants exhibited dose-dependent inhibitory properties. Along with the hyperproliferation of keratinocytes, an imbalance in apoptosis resulted in the manifestation of psoriasis, and the fractions demonstrated a significant reduction in mitochondrial membrane potential, suggesting the activation of the intrinsic apoptotic pathway [57,58,59]. Additionally, these fractions triggered phosphatidylserine externalization, further confirming their ability to induce apoptosis in HaCaT cells. Considering the role of the pro-inflammatory cytokines, IL-8 and RANTES, in the pathogenesis of psoriasis, we further investigated the inhibition in IL-8 and RANTES levels post-treatment [60,61]. As evidenced by their ability to downregulate IL-8 and RANTES secretion, these fractions exhibited promising anti-inflammatory activity.

Our primary aim was to investigate the most active fractions to narrow down the search of anti-psoriatic bioactive compound(s). Therefore, based on in vitro results, we selected the two most active fractions from each plant for further investigations into the IMQ- induced psoriasis mice model. The assessment of body weight and skin edema provided key insights into the therapeutic effects of the plant fractions. Clobetasol, the positive control, significantly reduced body weight and maintained minimal skin edema throughout the study, consistent with its well-documented anti-inflammatory properties [62]. In contrast, ASE2, ASE3, WTE2, and WTE3 fractions not only prevented severe weight loss but also maintained moderate edema, with WTE fractions showing a stronger effect, suggesting a more pronounced systemic response. The initial weight loss observed until day 3 in IMQ-induced psoriatic mice was likely due to systemic inflammation, increased metabolic demands, and psoriasis-related stress, in line with previous findings in inflammatory disease models [63,64]. However, the subsequent recovery of body weight in the ASE2, ASE3, WTE2, and WTE3 -treated groups indicates reduced inflammation and improved physiological balance, likely driven by the anti-inflammatory properties of these fractions [65]. In contrast, the disease control and clobetasol-treated groups failed to regain weight, which may be attributed to persistent inflammation or the prolonged effects of corticosteroids, known to suppress normal weight gain [62]. The ability of these fractions to mitigate weight loss and edema implies their potential to alleviate some of the physical symptoms associated with psoriasis.

The reduction in PASI scores confirmed the anti-psoriatic effects of the tested fractions, with WTE2 and WTE3 showing the most significant improvements, comparable to clobetasol, while ASE2 had a moderate effect and ASE3 was the least effective, suggesting the need for optimization. Fluctuations in PASI scores reflect the dynamic resolution of psoriasis rather than exacerbation, as psoriasis involves cyclic inflammation, keratinocyte hyperproliferation, and immune cell infiltration [66,67]. The initial PASI reduction followed by a slight increase may result from transient immune activation or delayed epidermal turnover before stable remission [68]. Clinical studies suggest that early immune responses to treatment precede lesion clearance [69], aligning with reports of non-linear recovery in psoriasis management [70]. The fluctuation in the PASI score over the treatment period should be carefully interpreted. In Figure 10, the increase in PASI scores after day 5 does not necessarily indicate that the treatments are exacerbating psoriasis. Instead, this trend could be due to the natural progression of IMQ-induced psoriatic lesions, where inflammation and skin thickening peak before resolution begins. Additionally, some treatments may have a delayed effect, where initial immune activation or skin remodeling processes contribute to temporary worsening before improvement. The subsequent decline in PASI scores in certain groups suggests that therapeutic effects take time to manifest. The observed fluctuations may also be influenced by variability in individual responses, differences in treatment efficacy, or transient immune responses triggered by the interventions.

Furthermore, the ear punch biopsy weights, which reflect local inflammation, revealed that WTE2 and WTE3 significantly reduced tissue inflammation, as evidenced by lower biopsy weights, comparable to clobetasol. Conversely, ASE3 did not significantly reduce ear punch biopsy weight, aligning with its lower efficacy observed in other parameters. The ability of WTE fractions to reduce tissue inflammation suggests that these extracts might modulate local inflammatory pathways effectively, a critical factor in the management of psoriasis. Spleen weight, a marker of systemic inflammation, was significantly reduced in the clobetasol-treated group but not in the groups treated with ASE and WTE fractions. This suggests that while these plant extracts effectively manage local skin inflammation, they may not exert a strong systemic anti-inflammatory effect, or their effects are more localized to the skin. Further studies are necessary to understand their mechanism of action and potential systemic benefits.

Additionally, histopathological analysis provided detailed insights into the effects of the fractions on skin structure. WTE2 and WTE3 significantly ameliorated psoriatic histopathological features, including hyperkeratosis, parakeratosis, and inflammatory cell infiltration, with histopathological scores comparable to clobetasol. ASE2 and ASE3, while showing some improvement, were less effective, particularly ASE3, which showed only modest histological improvements. These findings corroborate the observed trends in PASI scores and biopsy weights, highlighting WTE2 and WTE3 as promising fractions for further development.

The measurement of IL-23 and IL-17 levels provided molecular evidence of the anti-inflammatory effects of the plant fractions. Both WTE2 and WTE3 significantly reduced the serum and skin levels of these cytokines, similar to clobetasol, indicating their potential to modulate key inflammatory pathways involved in psoriasis. While ASE2 also demonstrated a significant reduction in IL-17 levels, ASE3 was less effective, particularly in the skin homogenate, aligning with its overall lower efficacy observed in other assays.

The collective data suggest that WTE2 and WTE3 fractions possess strong anti-psoriatic properties, potentially making them viable candidates for further development as natural therapeutic agents. ASE2 also shows promise but requires further optimization. The differential effects observed between the ASE and WTE fractions may be attributed to differences in their phytochemical composition, which warrants further investigation. Future studies should focus on elucidating the molecular mechanisms underlying the observed effects, particularly the pathways modulated by WTE2 and WTE3. Additionally, exploring the potential of combining these fractions with other therapeutic agents could enhance their efficacy. Long-term studies and clinical trials will be essential to confirm their safety and effectiveness in human populations.

Despite the promising results, this study has several limitations that should be considered. First, while the in vitro experiments using HaCaT cells provided valuable insights into the anti-proliferative and pro-apoptotic effects of the plant extract fractions, these findings may not fully translate to the complex biological environment in vivo. The HaCaT cell line, although a widely accepted model for psoriasis research, does not entirely replicate the intricate interactions occurring in human psoriatic skin. Additionally, the in vivo experiments using IMQ-induced psoriatic mice, while reflective of many aspects of human psoriasis, may not capture the full spectrum of disease pathology and immune responses seen in human patients. Moreover, the study focused on specific fractions obtained through a chloroform gradient system, which may not encompass all the bioactive compounds present in the crude extracts. Nevertheless, our findings collectively highlight the promising therapeutic potential of these fractions in managing psoriasis. By targeting the underlying inflammatory and immune processes involved in disease pathogenesis, these fractions address both local and systemic aspects of psoriasis. This multifaceted strategy has the potential to provide holistic improvement in disease symptoms and enhance patient quality of life. However, further studies involving more comprehensive profiling of the active components, as well as preclinical investigations, are necessary to confirm the therapeutic efficacy of these fractions and to better understand their mechanisms of action in treating psoriasis.

## 4. Material and Methods

### 4.1. Plant Materials and Preparation of Extracts

Leaves of *Alstonia scholaris* and *Wrightia tinctoria* were sourced from IMPCOPS, India authenticated and deposited at Amity University, Noida, UP, India for future reference with voucher specimen No.: 0001 for *A. scholaris*, and 0002 for *W. tinctoria.* High-performance liquid chromatography (HPLC) analysis of the plant extracts against their respective biomarker compounds was previously conducted [15]. The leaves were washed, dried, powdered, and extracted with 99% ethanol using an orbital shaker and Whatman filter paper. The solvent was evaporated using a vacuum rotary evaporator, and the dried residues were stored at 4 °C. The ethanol extracts were subjected to fractionation using silica gel chromatography with a chloroform gradient system. Six distinct fractions were prepared for each plant extract by varying the chloroform-to-methanol ratio as: 100:0, 80:20, 60:40, 40:60, 20:80, and 0:100 (*v*/*v*). Each fraction was collected and respectively named ASE1, ASE2, ASE3, ASE4, ASE5, and ASE6 for *A. scholaris*, and WTE1, WTE2, WTE3, WTE4, WTE5, and WTE6 for *W. tinctoria*. It is important to note that no residual ethanol was present in the final fractions, as the ethanol used for extraction was completely evaporated before fractionation. The working concentrations of the fractions were prepared in a manner ensuring no solvent-induced cytotoxicity. These fractions were tested for in vitro anti-psoriatic activity, and the most active fractions were selected for formulation and in vivo studies.

### 4.2. Chemical Characterization of the Crude Extracts

The chemical profiling of the extract was carried out using a Perkin Elmer Clarus 680 Gas Chromatograph (GC) coupled with a Clarus SQ 8C Mass Spectrometer (MS). The system was controlled by TurboMass software (version 6.1.1) and utilized the NIST-2014 spectral library for compound identification. Separation of components was achieved using a fused silica capillary column, HP-5MS (30.0 m × 0.25 mm ID × 0.25 μm df), with a stationary phase composed of 5% biphenyl and 95% dimethylpolysiloxane. Helium (He) served as the carrier gas at a constant flow rate of 2 mL/min. The injection port was set at 280 °C with a splitless injection of 1 μL of the extract. The oven temperature program was as follows: initial temperature of 100 °C for 2 min, ramped to 200 °C at 10 °C/min, held for 3 min, and then increased to 300 °C at 25 °C/min, where it was maintained for 10 min, resulting in a total run time of 29 min. The mass spectrometer operated in electron impact (EI) ionization mode at 70 eV, with an inlet line and ion source temperatures of 250 °C and 230 °C, respectively. The mass range was scanned from 40 to 600 Da, with a scan time of 0.2 s and an interval of 0.1 s. The acquired mass spectra were compared with known spectra from the NIST-2014 library to identify the chemical constituents present in the extract.

### 4.3. In Vitro Assessment

#### 4.3.1. Cell Culture

The HaCaT cell line, consisting of transformed immortal human epidermal keratinocytes, was obtained from ThermoFisher, Mumbai, India. These adherent cells were cultured in Dulbecco’s Modified Eagle Medium (DMEM) (Himedia, Mumbai, India) supplemented with 10% fetal bovine serum (FBS) (Gibco, New York, NY, USA), 100 U/mL penicillin (Gibco, New York, NY, USA), and 10 μg/mL streptomycin (Gibco, New York, NY, USA). The cultures were maintained in an incubator at 37 °C with a 5% CO_2_ atmosphere.

#### 4.3.2. Anti-Proliferative Assay

HaCaT cells were seeded at a density of 10,000 cells per well in a 96-well cell culture-grade plate and incubated overnight. The cells were then treated with chloroform-based fractions ASE1, ASE2, ASE3, ASE4, ASE5, ASE6, WTE1, WTE2, WTE3, WTE4, WTE5, and WTE6 at concentrations ranging from 0.1 μg/mL to 200 μg/mL for 24, 48, and 72 h. Curcumin (25 µM) was used as a positive control [71]. Cellular proliferation inhibition was assessed using the MTT assay (0.5 mL, Sigma-Aldrich, St. Louis, MO, USA) according to the manufacturer’s protocol, and absorbance was measured at 540 nm. The percentage of cellular proliferation inhibition was calculated using the formula: percent inhibition = ([Y − X]/Y) × 100, where X represents the absorbance of treated cells at 540 nm, and Y represents the absorbance of untreated control cells at 540 nm.

#### 4.3.3. Mitochondrial Depolarization Assay

The mitochondrial depolarization assay was performed as described in our previous study. HaCaT cells were treated with various concentrations (0.1 µg/mL, 1 µg/mL, 10 µg/mL, 100 µg/mL, 200 µg/mL) of the chloroform-based fractions ASE1, ASE2, ASE3, ASE4, ASE5, ASE6, WTE1, WTE2, WTE3, WTE4, WTE5, and WTE6 for 48 h, along with curcumin (25 µM) as a positive control. Following treatment, the cells were incubated with JC-1 dye to assess mitochondrial membrane potential (MMP). The change in MMP (ΔψM) was quantified as the ratio of red to green fluorescence intensities. The percentage decrease in MMP was calculated to determine the apoptotic effect. HaCaT cells were cultured in 96-well plates (10,000 cells per well) and exposed to different concentrations of ASE1, ASE2, ASE3, ASE4, ASE5, ASE6, WTE1, WTE2, WTE3, WTE4, WTE5, and WTE6 (5 μg/mL, 10 μg/mL, 50 μg/mL, and 100 μg/mL) or curcumin (25 µM) for 48 h. After treatment, the supernatant was removed and the cells were incubated with 100 µL of JC-1 dye (EMD Millipore) in phosphate-buffer saline (PBS) for 30 min at 37 °C in a humidified incubator with 5% CO_2_. The supernatant was then removed, and the cells were washed twice with PBS. Finally, 100 µL of PBS was added to each well, and the red fluorescence (excitation 550 nm, emission 600 nm) indicative of healthy mitochondria and the green fluorescence (excitation 485 nm, emission 535 nm) indicative of decreased membrane potential were measured using a Biotek Synergy HT plate reader. MMP was calculated as the ratio of red fluorescence intensity (healthy mitochondria) to green fluorescence intensity (apoptotic cells). The mitochondrial depolarization or apoptotic effect was quantified using the following formula:

% MMP Decrease = ([R − X]/R) × 100

where X represents the ΔψM for treated cells, and R represents the ΔψM for untreated control cells [59,72].

#### 4.3.4. Enzyme-Linked Immunosorbent Assay (ELISA) for Inflammatory Markers

HaCaT cells were initially cultured in DMEM supplemented with 10% FBS for 24 h, followed by serum deprivation (0.1% FBS) for an additional 24 h. After serum deprivation, the cells were treated with various concentrations (ranging from 5 μg/mL to 100 μg/mL) of ASE1, ASE2, ASE3, ASE4, ASE5, ASE6, WTE1, WTE2, WTE3, WTE4, WTE5, and WTE6 for another 24 h. Curcumin, at 25 µM, served as a positive control. Following pretreatment, the cells were stimulated with human TNF-α (10 ng/mL, R&D Systems, Minneapolis, MN, USA) and incubated for an additional 24 h. The supernatants from the cell cultures were collected and analyzed for IL-8 and RANTES levels using ELISA kits according to the manufacturer’s protocol (R&D Systems, Minneapolis, MN, USA). Absorbance was measured at 540 nm, and the percentage inhibition of secreted pro-inflammatory markers in the culture supernatants was calculated using the formula:

% inhibition = ([B − A]/B) × 100

where A represents the concentration of the marker secreted by TNF-α-stimulated cells treated with the specified plant extract, and B represents the concentration of the marker secreted by TNF-α-stimulated untreated control cells [73].

### 4.4. In Vivo Analysis

#### 4.4.1. Mice

Female BALB/c mice (10–12 weeks) were purchased from Hylasco Biotechnology Pvt, Ltd., India. The mice were allowed to acclimatize to the environment for 7 days before the commencement of the experiments. The pathogen-free animals were maintained under standard pathogen-free conditions (12 h light and dark cycle each at an ambient temperature of 19.1 to 21.6 °C and a relative humidity of 42–63%). They were fed with conventional feed purchased from Advanced research and analytical services, Ghaziabad, India. The feed was provided ad libitum. Filtered drinking water was also provided ad libitum. Corn cob was used as bedding material. The test facility was registered (Registration No. 64/PO/RcBi/S/99/CPCSEA) for experiments on animals with the Committee for the Control and Supervision of Experiments on Animals (CCSEA), Ministry of Environment and Forest, Govt. of India. The experimental protocol (IAEC Protocol No.: IAEC/87/1691) was approved by the Institutional Animal Ethics Committee (IAEC) of Dabur Research Foundation, India. The animals were randomized based on body weight and grouped into seven groups (G1 to G7) as shown in Table 3.

#### 4.4.2. Preparation of Test Item Formulation/Hydrogels

Hydrogels were prepared by soaking 0.6 g of Carbomer Ultrez 20 polymer in distilled water for 2 h and the final formulation amount was adjusted to 200 g. The formulation was then neutralized using triethanolamine (TEA) to achieve a gel texture. Subsequently, 0.5 g of the fractions ASE2, ASE3, WTE2, WTE3 were incorporated into 50 g of the gel base to prepare four individual sets, and each mixture was homogenized to ensure uniform distribution of the active components.

#### 4.4.3. Psoriasis Induction, Body Weight, Edema, and Spleen Weight Assessment

Forty-two animals were randomized based on body weight and grouped into seven groups (G1 to G7), as shown in Table 3. Each group comprised six animals. All mice had their backs shaved. In group 1, the vehicle cream, propylene glycol, was applied to the shaved backs and served as the normal control group. Skin inflammation in mice was induced by daily application of a dose of approximately 63 mg of IMQ (Imiquimod) cream (Glenmark Pharmaceuticals 5%) (containing 3.125 mg of IMQ) [74] on shaved dorsal back area (2.5 × 2 sq. cm) of the mice of group 2 to group 7 for 8 days. Group 3 was treated topically with the reference drug, Clobetasol (GlaxoSmithKline, Brentford, London, UK), at a dose of 100 mg (0.1%), and served as the positive control group. G4 to G7 were treated topically with the gel formulations of ASE2, ASE3, WTE2, and WTE3, respectively, at a dose of 250 mg. The test items were applied topically daily on the shaved area for 8 days. A two-hour gap was maintained between the dosing of the inducing agent and the test item or reference drug. The body weight of all the animals was recorded daily. Ear thickness was measured using a digital caliper (MITUTOYO) on Day 0, Day 2, Day 4, Day 6, and Day 8 for all the experimental groups. The change in ear thickness (edema) was calculated in comparison with basal ear thickness to determine the inflammation caused by IMQ. On Day 9, the animals were sacrificed, and 4 mm punch ear biopsies were taken and weighed. After the ear skin biopsy weight was taken, the spleens from all the animals were isolated and weighed to assess splenomegaly.

#### 4.4.4. Scoring Severity Based on Erythema and Scaling

To score the severity of skin inflammation in mice, the Psoriasis Area and Severity Index (PASI) were calculated. The exposed areas of the mouse’s back were evaluated for erythema, scale, and thickening using a 4-point system (where 0 = no clinical signs, 1 = slight clinical signs, 2 = moderate clinical signs, 3 = marked clinical signs and 4 = very marked clinical signs.) The cumulative score (erythema plus scaling plus thickening) served as a measure of the severity of inflammation.

#### 4.4.5. Histopathological Analysis

On Day 9, all animals were sacrificed via CO_2_ asphyxiation, and the dorsal skin was surgically excised and collected. The tissues were fixed in 10% neutral buffered formalin by the immersion method. The fixed tissues were processed in different grading of alcohol and xylene by an automatic spin tissue processor (Thermo Scientific—STP120-3, Waltham, MA, USA) and routine paraffin embedding (5 μm thick) was performed by a Tissue Embedding and Cold module- Thermo Scientific- Histostar, followed by sectioning (Semi-Automatic Microtome-Leica—RM2245 [3–6 micron]). The sections were stained with hematoxylin and eosin. The prepared slides were examined under a microscope to record histopathological findings such as the extent of lesions, severity of hyperkeratosis, number and stage of pustules, height of epidermal hyperplasia, and the degree of inflammation in the dermal and subcutaneous tissues. The slide imaging was carried out by Radical ProCam version 4.8 Software.

Histopathological scoring was provided to assess the severity of tissue abnormalities, typically categorized as 0 for no apparent abnormalities (NAD), 1 for mild, 2 for moderate, and 3 for severe manifestations of histopathological features.

#### 4.4.6. Inflammation Detection by ELISA

On day 9, all the animals were anesthetized, blood was collected for the serum separation and stored for further biomarker analysis. Skin tissue was homogenized in 1× PBS and centrifuged at 10,000× *g* at 4 °C at the concentration of 10% (*w*/*v*). Both serum and skin samples were analyzed for inflammatory cytokines such as IL-17 and IL-23 using GENLISA™ Mouse ELISA (Krishgen Biosystems, Mumbai, India) following the manufacturer’s instructions.

### 4.5. Statistical Analysis

The experimental data were presented as mean ± standard error of the mean (SEM), and all values were normalized relative to the control group for the vitro experiments. For the vivo experiments, all data were expressed in mean ± SEM and analyzed by one-way and two-way ANOVA followed by Dunnett’s test and/or the Bonferroni test for statistical significance.

## 5. Conclusions

This study provides a comprehensive evaluation of the anti-psoriatic potential of fractionated extracts from *Alstonia scholaris* and *Wrightia tinctoria*. Our findings demonstrate that specific fractions, particularly ASE3 from *A. scholaris* and WTE2 and WTE3 from *W. tinctoria*, exhibit significant anti-proliferative, pro-apoptotic, and anti-inflammatory effects both in vitro and in vivo. These fractions effectively reduced key markers of psoriasis, such as epidermal hyperplasia, immune cell infiltration, and pro-inflammatory cytokine levels, in an IMQ-induced psoriatic mice model. The results highlight the potential of these plant fractions as novel therapeutic agents for the treatment of psoriasis, offering an alternative to conventional therapies. The ability of these fractions to modulate both local skin inflammation and systemic immune responses emphasize their promise in providing the comprehensive management of psoriasis.

Future research should focus on identifying the active constituents within these fractions and their mechanism of action and pharmacokinetic analysis, optimizing their formulation for topical or systemic administration, and conducting clinical trials to validate their efficacy and safety in human subjects. Overall, this study contributes to the growing body of evidence supporting the use of medicinal plants in dermatology and paves the way for the development of new treatments for psoriasis.

## Figures and Tables

**Table 1:** The table represents the number of chemical compounds identified through GC–MS analysis.

RT	Compound Name	Molecular Formula	Molecular Weight
12.43	Oxirane	C_12_H_24_O	184.182715
	Tetradecanal	C_14_H_28_O	212.214016
	Pentadecanal	C_15_H_30_O	226.229666
	Vinyl lauryl ether	C_14_H_28_O	212.214016
	Dodecanal	C_12_H_24_O	184.182715
	Undecane	C_10_H_16_N_2_O	180.126264
	Hexadecanal	C_16_H_32_O	240.245316
	Pentadecanal	C_15_H_30_O	226.229666
	Hexadecanal	C_16_H_32_O	240.245316
	1,2-Epoxyundecane	C_11_H_22_O	170.167066
13.48	1-Ethoxypentan-3-ol	C_7_H_16_O_2_	132.115029
	Hexanol	C_7_H_16_O	116.1201153
	*N*-Acetyl-d-threo-*O*-methylthreonine	C_7_H_13_NO_4_	175.084458
	2-Butene	C_8_H_16_O_2_	144.115029
	Silane	C_8_H_20_Si	144.133428
	2-Acetylamino-3-hydroxy-propionic acid	C_5_H_9_NO_4_	147.053158
	Oxirane	C_6_H_12_O_2_	116.0837297
	Heptane	C_7_H_14_O	114.104465
	Oxirane	C_5_H_10_O_2_	102.0680795
	Rhamnitol	C_8_H_14_O_5_	190.084124
14.94	Aziridine	C_6_H_14_N_2_	114.1156983
	Tris(aziridinomethyl)hydrazine	C_9_H_19_N_5_	197.164045
	5-Aziridinopentanol	C_7_H_15_NO	129.115364
	5-Ethyl-3-nonanol	C_11_H_24_O	172.182715
	Quinolinedione	C_25_H_38_N_2_O_3_	414.288242
	Methyl 5-piperidino-4-ketocaproate	C_11_H_19_NO_3_	213.136494
	Oxirane	C_11_H_22_O_2_	186.16198
	1,3-Diethoxy-2-methylenepropane	C_8_H_16_O_2_	144.115029
	2-Butene	C_8_H_16_O_2_	144.115029
15.11	1,3-Dioxane-2-propanol	C_8_H_16_O_3_	160.109944
	Carboxylic acid	C_4_H_6_O_3_	102.031694
	Hexanol	C_7_H_16_O	116.1201153
	Dimethyldiaziridine	C_7_H_17_N_3_	143.142247
	Oxirane	C_5_H_10_O_2_	102.0680795
	1-Tetradecanamine	C_14_H_31_N	213.24565
	Dimethyldiaziridine	C_7_H_14_N_2_O	142.110613
	4-Oxopentyl formate	C_6_H_10_O_3_	130.062994
	Heptanol	C_9_H_20_O	144.151415
	Heptane	C_7_H_14_O	114.104465

**Table 2:** The table represents identification of chemical compounds in the ethanolic extract of *Wrightia tinctoria* based on retention times (RT) during chromatographic analysis. Multiple compounds were detected at distinct RTs, characterized by their unique chromatographic profiles.

RT	Compound Name	Molecular Formula	Molecular Weight
12.48	*N*-[4-Aminobutyl]aziridine	C_6_H_14_N_2_	114.11
	1,4,5,8-Tetraazadecalin	C_6_H_14_N_4_	142.12
	Methyldicyanophosphine	C_3_H_3_N_2_P	98
	5-Aziridinopentanol	C_7_H_15_NO	129.11
	1,4-Butanediamine, *N*-(3-aminopropyl)	C_7_H_19_N_3_	145.15
	1,3-Diethoxy-2-methylenepropane	C_8_H_16_O_2_	144.11
	Betaine	C_5_H_11_NO_2_	117.07
	Tris(aziridinomethyl)hydrazine	C_9_H_19_N_5_	197.16
	4-Methyl-2-hexanol	C_7_H_16_O	116.12
	3-Ethyl-2-heptanol	C_9_H_20_O	144.15
13.44	4(1H)-Pyridinone, 2,3-dihydro-1-methyl	C_6_H_9_NO	111.06
	Pyrazol-4-amine	C_5_H_9_N_3_	111.07
	2(1H)-Pyridinone, 5-hydroxy	C_5_H_5_NO_2_	111.03
	2(1H)-Pyridinone, 3-hydroxy	C_5_H_5_NO_2_	111.03
	*N*-Methylmaleimide	C_5_H_5_NO_2_	111.03
	Tropinone, 6β-methoxy-, (+)	C_9_H_15_NO_2_	169.11
	Quinuclidine	C_7_H_13_N	111.1
14.86	(*E*)-Tetradec-2-enal	C_14_H_26_O	210.19
	Vinyl lauryl ether	C_14_H_28_O	212.21
	6-Dodecanol acetate	C_14_H_28_O_2_	228.2
	13-Methyltetradecanal	C_15_H_30_O	226.22
	Hexadecane, 1-(ethenyloxy)	C_18_H_36_O	268.27
	3,7,11-Trimethyldodecylacetate	C_17_H_34_O_2_	270.25
	(E)-Hexadec-2-enal	C_16_H_30_O	238.22
	2-Tridecenal, (E)	C_13_H_24_O	196.18
	1-Eicosanol	C_20_H_42_O	298.32
	Tridecanal	C_13_H_26_O	198.19
14.99	(3S,5R,8aR)-3-(Hex-5-en-1-yl)-5-(pent-4-en-1-yl)octahydroindolizine	C_19_H_33_N	275.26
	(5R,8aR)-3-(Hex-5-en-1-yl)-5-(pent-4-en-1-yl)octahydroindolizine	C_19_H_33_N	275.26
	1-Methyl-3-[4-(1-trimethylsilyloxyethylidene)-cyclohexa-3,5-dienylidene]-triazene	C_12_H_19_N_3_OSi	249.12
	6-Azaspiro[2.5]octa-4,7-diene-6-carboxylic acid, 2,2-dimethyl-, ethyl ester	C_12_H_17_NO_2_	207.12
	Benzoic acid, 3-(diethylamino)-, methyl ester	C_12_H_17_NO_2_	207.12
	Piperidine-2,6-dione, 2-oxo-2-[6-oxo-2-(1-pyrrolidinyl)cyclohexenyl]ethyl	C_17_H_22_N_2_O_4_	318.15
	Pyrrolidine, *N*-(menth-3-en-3-yl)	C_14_H_25_N	207.19
	3-(6,7-Dimethoxy-3,4-dihydro-1H-isoquinolin-2-yl)propan-1-amine	C_14_H_22_N_2_O_2_	250.16
	(+)-Salsolidine	C_12_H_17_NO_2_	207.12
	2,9-Dimethyl-4-ethynyl-trans-decahydroquinol-4-ol	C_13_H_21_NO	207.16

**Table 3:** The table represents allocation of animals for experiments.

Groups	Treatment	Dose, Regimen and Route of Administration	Animals per Group
G1: Normal Control	Vehicle	250 mg, qdx8, Topical	6
G2: Psoriasis Control	IMQ + Vehicle	~63 mg, qdx8, Topical + 250 mg, qdx8, Topical	6
G3: Reference Item	IMQ + Clobetasol	~63 mg, qdx8, Topical + 100 mg (0.1%), qdx8, Topical	6
G4: Test Item Treatment-1	IMQ + ASE2	~63 mg, qdx8, Topical + 250 mg, qdx8, Topical	6
G5: Test Item Treatment-2	IMQ + ASE3	~63 mg, qdx8, Topical + 250 mg, qdx8, Topical	6
G6: Test Item Treatment-3	IMQ + WTE2	~63 mg, qdx8, Topical + 250 mg, qdx8, Topical	6
G7: Test Item Treatment-4	IMQ + WTE3	~63 mg, qdx8, Topical + 250 mg, qdx8, Topical	6

## Data Availability

Data will be made available on request.

## Abbreviations

ANOVA	Analysis of variance
ASE1	Fraction Prepared in 100% Chloroform of Ethanolic Extract of *Alstonia scholaris*
ASE2	Fraction Prepared in 80% Chloroform and 20% Methanol of Ethanolic Extract of *Alstonia scholaris*
ASE3	Fraction Prepared in 60% Chloroform and 40% Methanol of Ethanolic Extract of *Alstonia scholaris*
ASE4	Fraction Prepared in 40% Chloroform and 60% Methanol of Ethanolic Extract of *Alstonia scholaris*
ASE5	Fraction Prepared in 20% Chloroform and 80% Methanol of Ethanolic Extract of *Alstonia scholaris*
ASE6	Fraction Prepared in 100% Methanol of Ethanolic Extract of *Alstonia scholaris*
CCSEA	Committee for Control and Supervision of Experiments on Animals
DMEM	Dulbecco’s modified Eagle’s medium
DMSO	Dimethyl Sulphoxide
E	Ethanolic extract
ELISA	Enzyme-linked immunosorbent assay
FBS	Fetal bovine serum
HPLC	High-performance liquid chromatography
IAEC	Institutional Animal Ethics Committee
IMQ	Imiquimod
IL-8	Interleukin-8
IL-23	Interleukin-23
IL-17	Interleukin-17
JC-1	5,5,6,6′-tetrachloro- 1,1′,3,3′-tetraethylbenzimi-dazoylcarbocyanine iodide
MMP	Mitochondrial membrane potential
MTT	3-[4,5-dimethylthiazol-2-yl]-2,5 diphenyl tetrazolium bromide
PASI	Psoriatic area severity index
PBS	Phosphate-buffered saline
Qd	Quaque die (daily)
RANTES	Regulated upon activation, normal T Cell expressed and presumably secreted
SEM	Standard error of the mean
TNF-α	Tumor necrosis factor alpha
WTE1	Fraction Prepared in 100% Chloroform of Ethanolic Extract of *Wrightia tinctoria*
WTE2	Fraction Prepared in 80% Chloroform and 20% Methanol of Ethanolic Extract of *Wrightia tinctoria*
WTE3	Fraction Prepared in 60% Chloroform and 40% Methanol of Ethanolic Extract of *Wrightia tinctoria*
WTE4	Fraction Prepared in 40% Chloroform and 60% Methanol of Ethanolic Extract of *Wrightia tinctoria*
WTE5	Fraction Prepared in 20% Chloroform and 80% Methanol of Ethanolic Extract of *Wrightia tinctoria*
WTE6	Fraction Prepared in 100% Methanol of Ethanolic Extract of *Wrightia tinctoria*
µg	Microgram
µM	Micromolar
µL	Microliter
mg	Milligram
mL	Milliliter
mM	Millimolar
nm	Nanometer
mg/kg	milligram/kilogram
mm	millimeter
cm	Centimeter
°C	Degree Centigrade
%	Percentage
